# ‘*Candidatus* Phytoplasma solani’ interferes with the distribution and uptake of iron in tomato

**DOI:** 10.1186/s12864-019-6062-x

**Published:** 2019-09-10

**Authors:** Sara Buoso, Laura Pagliari, Rita Musetti, Marta Martini, Fabio Marroni, Wolfgang Schmidt, Simonetta Santi

**Affiliations:** 1 Department of Agricultural, Food, Environmental and Animal Sciences, University of Udine, Via delle Scienze 206, 33100 Udine, Italy; 2 IGA Technology Services, Via Jacopo Linussio, 51, 33100 Udine, Italy; 3 Institute of Plant and Microbial Biology, Academia Sinica, 11529 Taipei, Taiwan; 4 Biotechnology Center, National Chung Hsing University, 40227 Taichung, Taiwan

**Keywords:** Iron deficiency, Leaves, Porphyrin, Chlorophyll, Carotenoids metabolism, Phytoplasma, Phloem, Roots, Tomato, NGS

## Abstract

**Background:**

‘*Candidatus* Phytoplasma solani’ is endemic in Europe and infects a wide range of weeds and cultivated plants. Phytoplasmas are prokaryotic plant pathogens that colonize the sieve elements of their host plant, causing severe alterations in phloem function and impairment of assimilate translocation. Typical symptoms of infected plants include yellowing of leaves or shoots, leaf curling, and general stunting, but the molecular mechanisms underlying most of the reported changes remain largely enigmatic. To infer a possible involvement of Fe in the host-phytoplasma interaction, we investigated the effects of ‘*Candidatus* Phytoplasma solani’ infection on tomato plants (*Solanum lycopersicum* cv. Micro-Tom) grown under different Fe regimes.

**Results:**

Both phytoplasma infection and Fe starvation led to the development of chlorotic leaves and altered thylakoid organization. In infected plants, Fe accumulated in phloem tissue, altering the local distribution of Fe. In infected plants, Fe starvation had additive effects on chlorophyll content and leaf chlorosis, suggesting that the two conditions affected the phenotypic readout via separate routes. To gain insights into the transcriptional response to phytoplasma infection, or Fe deficiency, transcriptome profiling was performed on midrib-enriched leaves. RNA-seq analysis revealed that both stress conditions altered the expression of a large (> 800) subset of common genes involved in photosynthetic light reactions, porphyrin / chlorophyll metabolism, and in flowering control. In Fe-deficient plants, phytoplasma infection perturbed the Fe deficiency response in roots, possibly by interference with the synthesis or transport of a promotive signal transmitted from the leaves to the roots.

**Conclusions:**

**‘***Candidatus* Phytoplasma solani’ infection changes the Fe distribution in tomato leaves, affects the photosynthetic machinery and perturbs the orchestration of root-mediated transport processes by compromising shoot-to-root communication.

**Electronic supplementary material:**

The online version of this article (10.1186/s12864-019-6062-x) contains supplementary material, which is available to authorized users.

## Background

Phytoplasmas are plant pathogenic bacteria belonging to the class *Mollicutes*, a group of wall-less and pleomorphic microorganisms [1], which live a trans-kingdom parasitic life, infecting both plants and phloem-feeding insect hosts [2]. Phytoplasmas are associated with devastating damage to over 700 plant species worldwide, including many economically important crops, fruit trees, and ornamental plants [3, 4]. In infected plants, phytoplasmas reside in sieve elements of the phloem [5]. Phytoplasmas possess the smallest genome of any plant pathogenic bacteria (530–1350 kb), which is believed to have evolved from an ancestor via genomic reduction and fusion [6, 7], possibly as an adaptation to a nutrient-rich environment. The phytoplasma genome lacks genes encoding proteins involved in essential metabolic pathways such as the biosynthesis of amino fatty acids, the tricarboxylic acid cycle, and oxidative phosphorylation [6, 8–13]. Thus, phytoplasmas have strongly reduced metabolic capabilities and must absorb essential compounds from their hosts. This observation is supported by the presence of multiple copies of transport-related genes such as malate, metal-ion, and amino acid transporters in the phytoplasma genome [14]. Moreover, phytoplasmas secrete effectors that may directly interact, manipulate, or weaken their hosts [15, 16].

Phytoplasma-infected plants often exhibit a variety of symptoms, including virescence, phyllody, witches’-broom growth (proliferation of auxiliary or axillary shoots), abnormal elongation of internodes, flower malformation, and sterility. At the ultrastructural level, infected plants show occlusions in sieve elements due to phloem-protein agglutination and callose deposition which impair phloem mass flow [17] and often result in hyperplasia, necrosis, and the collapse of sieve elements [18–22]. Also, photosynthesis appears to be heavily affected in many phytoplasma-infected plants [23–29]. Several genes encoding photosystem I subunits and other components of the electron transport chain were found to be repressed by the infection [23, 24, 29]. Besides photosynthesis, the activities of key enzymes of the flavonoid and stilbene biosynthetic pathways, defence-related genes, and hormone-signalling pathway are modulated by the infection [23, 24, 26, 29–31]. In addition, yellowing of leaves or shoots, leaf curling, and general stunting are typical symptoms of infected plants, often associated with reduced content of chlorophyll, carotenoids, and proteins of light-harvesting complexes (LHC) [32, 33]. In grapevine, ‘*Candidatus* Phytoplasma solani’ (Ca. *P. solani*) infection was shown to inhibit sucrose phloem loading and to increase sucrose cleavage activity at the transcriptional level, causing a switch of leaf function from a source to a sink for carbohydrates [19, 30]. Some symptoms represent a derailment of programmed meristem fate and a modified pattern of growth due to pathogen-affected key meristem switching genes [34, 35]; the molecular mechanisms underlying most of the reported changes remain, however, largely enigmatic.

In host–pathogen interactions, competition for Fe is a determinant for an effective immune system and can affect susceptibility and resistance to a pathogen [36–38]. Although abundantly present in earth’s crust, the bioavailability of Fe to plants is restricted due to the poor solubility of Fe hydroxides that control Fe activity in aerated soils [39]. Plants have evolved complex, phylogenetically separated strategies to acquire Fe from soils [40]. All non-grass species, including tomato, employ a reduction-based Fe acquisition mechanism (Strategy I), in which Fe^3+^ is reduced by a Fe^3+^-chelate reductase (FRO2 in Arabidopsis, FRO1 in tomato) [41, 42]. The reduced Fe^2+^ is then transported across the plasma membrane by the transporter IRT1 [43, 44]. Solubilisation of scarcely available Fe pools in soil is supported by P-type ATPase-driven proton extrusion (AHA2 in Arabidopsis) [45]. Graminaceous species, on the other hand, rely on the secretion of Fe^3+^-binding phytosiderophores that are taken up after binding to Fe^3+^ without prior reduction of Fe, a strategy that is thought to be less pH dependent than the reductive Fe uptake adopted by non-grass species (Strategy II) [40]. Similar to grasses, Arabidopsis and other non-graminaceous species secrete Fe^3+^-mobilizing compounds such as flavins and coumarins [46–51]. In contrast to grasses, in Strategy I species reduction of the mobilized Fe^3+^ prior to uptake is obligatory [52].

The uptake of Fe is controlled by a complex interplay of regulatory proteins. The basic helix-loop-helix (bHLH) transcription factor FER in tomato and its Arabidopsis ortholog FER-LIKE IRON DEFICIENCY INDUCED TRANSCRIPTION FACTOR (FIT) emerged as the central regulator of Fe uptake [42, 53–56]. Upon Fe deprivation, FIT is activated in roots at the transcriptional and post-translational level, and forms heterodimers with members of subgroup lb. bHLH proteins (bHLH038/039/100/101). Similarly, in tomato FER interacts with SlbHLH068 to regulate Fe uptake genes [57–60]. In Arabidopsis, FIT heterodimers activate a suite of downstream genes such as *AHA2*, *FRO2*, and *IRT1* as well as genes involved in the secretion of Fe-mobilizing coumarins [52, 53, 57, 59, 61, 62]. The bHLH protein POPEYE positively regulates a group of genes that, vice versa, are negatively regulated by the E3 ubiquitin-protein ligase BRUTUS [63, 64]. This dual regulation seems to be critical to avoid overload of Fe [63]. Transcriptional activation of the Fe deficiency response in both strategies is dependent on the presence of IRON MAN, a family of peptides that accumulate in leaves and roots of Fe-deficient plants and control the transcription of a large suite of Fe homeostasis genes including FIT [65]. Interestingly, induced systemic resistance and Fe uptake share signalling components such as the Myb-type transcription factor MYB72 [66], indicative of a close interconnection of the two processes.

In the present study, we evaluated a possible involvement of Fe in the interaction between ‘*Ca*. *P. solani*’, a phytoplasma belonging to the 16SrXII group associated with stolbur disease [67], and tomato plants (Micro-Tom cultivar) as hosts. ‘*Ca*. *P. solani*’ is endemic in Europe and infects a wide range of weeds and cultivated plants reviewed in [68–72]. Our data are consistent with a model in which phytoplasma competes for Fe and perturbs the long-distance signalling of Fe status that is transmitted to the roots.

## Results

### Iron deficiency and phytoplasma infection induce leaf chlorosis

**Fig. 1:**
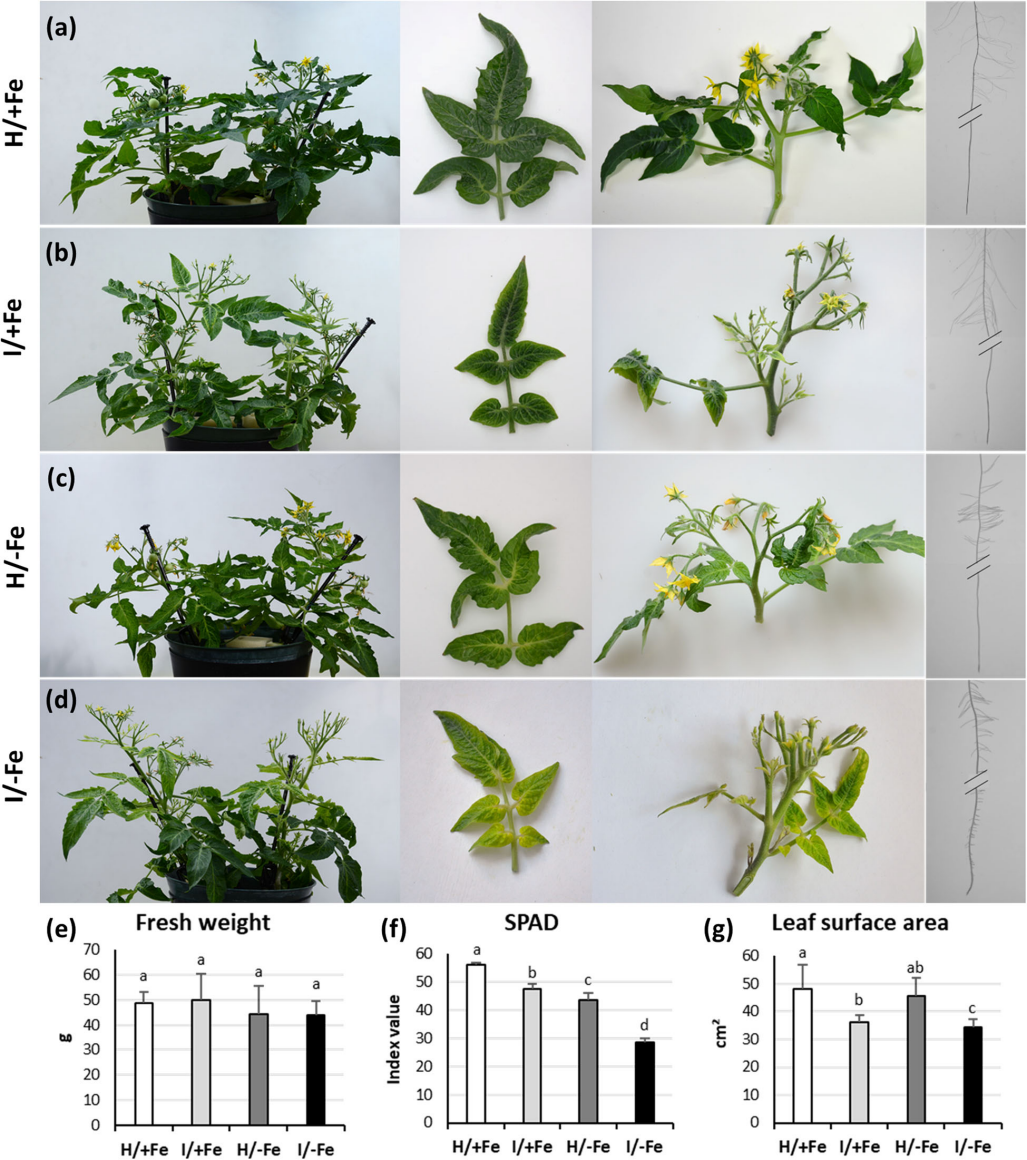
Phenotypes of representative tomato plants grown under different experimental conditions. **a**-**d** Whole plants, leaves, shoots, and roots of (**a**) healthy Fe-sufficient (H/+Fe) plants, **b** infected Fe-sufficient (I/+Fe) plants (**c**) healthy Fe-deficient (H/−Fe) plants, and (**d**) infected Fe-deficient plants, (I/−Fe). **e** Total plant fresh weight. Results are expressed as mean ± SD (*n* = 6). **f** Leaf SPAD index values of fully expanded leaves. Results are expressed as mean ± SD (*n* = 150). **g** Leaf surface area. Results are expressed as mean ± SD (*n* = 30). Different letters indicate statistically significant differences (*P* < 0.05) among conditions (one-way ANOVA followed by Holm-Sidak’s test)

Plant responses to Fe starvation, phytoplasma-infection, and phytoplasma-infection concurrent with Fe starvation were first studied considering whole plant morphology and plant biometric parameters (Fig. 1). Plants were analysed 5 weeks after grafting, when typical symptoms developed in both phytoplasma-infected and Fe-starved plants (Fig. 1). Symptoms of infected plants grown on Fe-replete media (I/+Fe plants) included swollen flower buds and malformed flowers with green petals (Fig. 1). Infected plants developed leaf chlorosis and decreased SPAD values, which quantify leaf light transmittance and indirectly chlorophyll content (Fig. 1b, f). Infected plants produced smaller leaves with reduced leaf area when compared to healthy plants (Fig. 1b, g). Root morphology remained unaffected by the infection (Fig. 1b). Non-infected Fe-deficient (H/−Fe) plants developed interveinal chlorosis on young leaves, which did not differ in size from leaves of control plants (Fig. 1c, f, g). Roots formed short lateral roots, extra root hairs, and swollen tips (Fig. 1c). No alterations were observed in shoot and flowers (Fig. 1c). Upon Fe-starvation, infected plants (I/−Fe plants) developed symptoms of both stresses, i.e. yellowing and surface reduction of leaves, the typical phytoplasma-induced alterations of shoots and flowers, as well as the root modifications caused by Fe deficiency (Fig. 1d, f, g). Notably, the combination of the two stresses intensified the chlorosis symptoms with interveinal chlorosis appearing together with yellowing of the leaf edges (Fig. 1d). Phytoplasma infection and Fe starvation had additive effects on the SPAD value (Fig. 1f). While plant morphology was severely affected by both Fe deficiency and phytoplasma infection, plant weight was not significantly altered by either treatment (Fig. 1e).

**Fig. 2:**
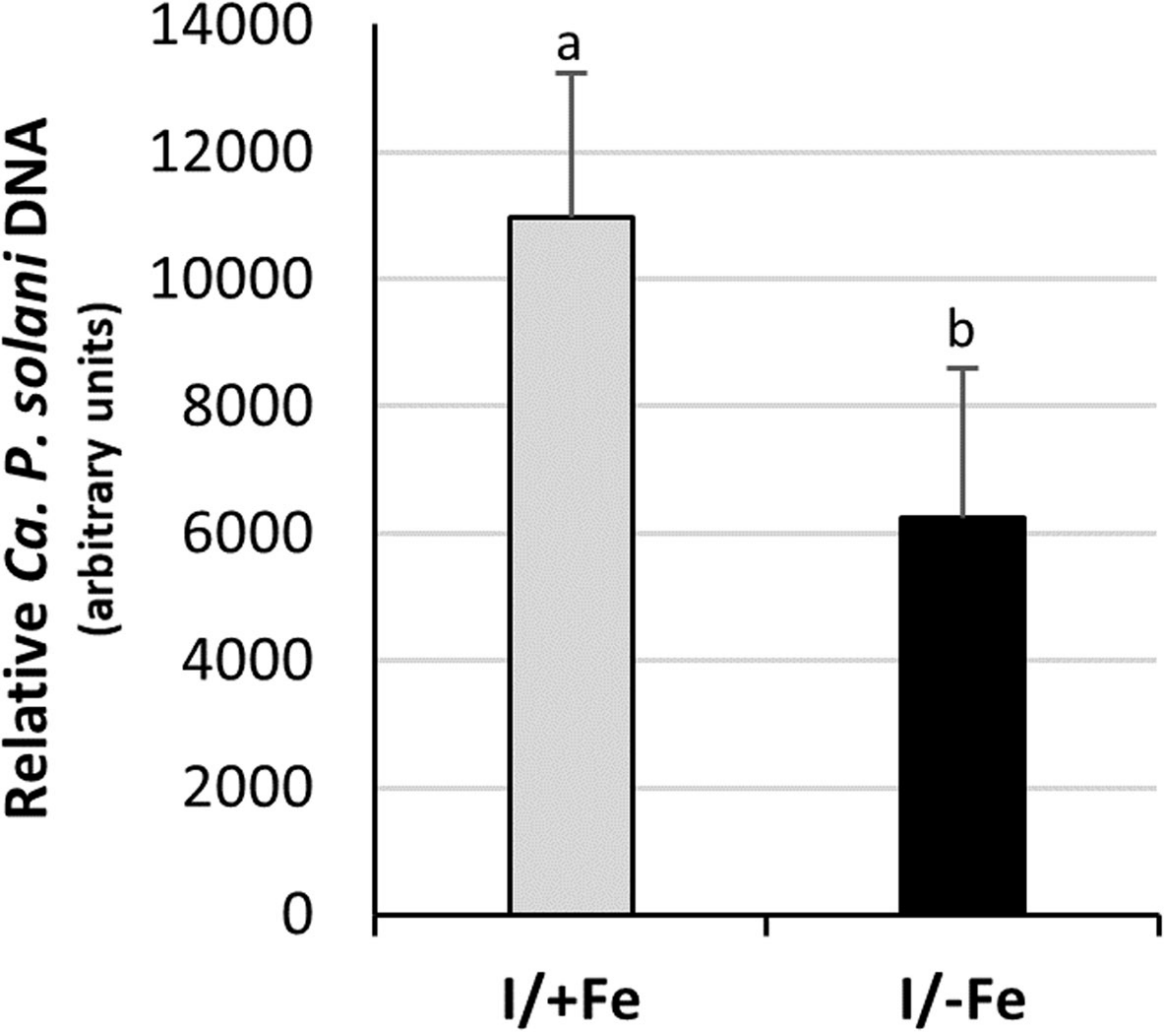
Relative quantification of ‘*Ca*. *P. solani*’ in infected Fe-sufficient and Fe-deficient tomato leaves. The amount of ‘Ca. *P. solani*’ DNA was determined by qPCR analysis of the *16SrRNA* gene of ‘Ca. *P. solani*’ relative to the tomato single-copy gene *Chloronerva*. Results are expressed as mean ± SD (*n* = 8). Different letters indicate statistically significant differences (*P* < 0.05) among conditions (one-way ANOVA followed by Holm-Sidak’s test)

To investigate the impact of Fe deficiency on pathogen replication capability, phytoplasma titre was quantified by qPCR in eight I/+Fe and eight I/−Fe plants (Fig. 2). In leaves of I/−Fe plants, the amount of phytoplasma was 1.7-fold reduced compared to leaves of I/+Fe, indicating that a healthy Fe status of the host supports growth of the pathogen.

### Iron deficiency and phytoplasma infection alter chloroplast ultrastructure

**Fig. 3:**
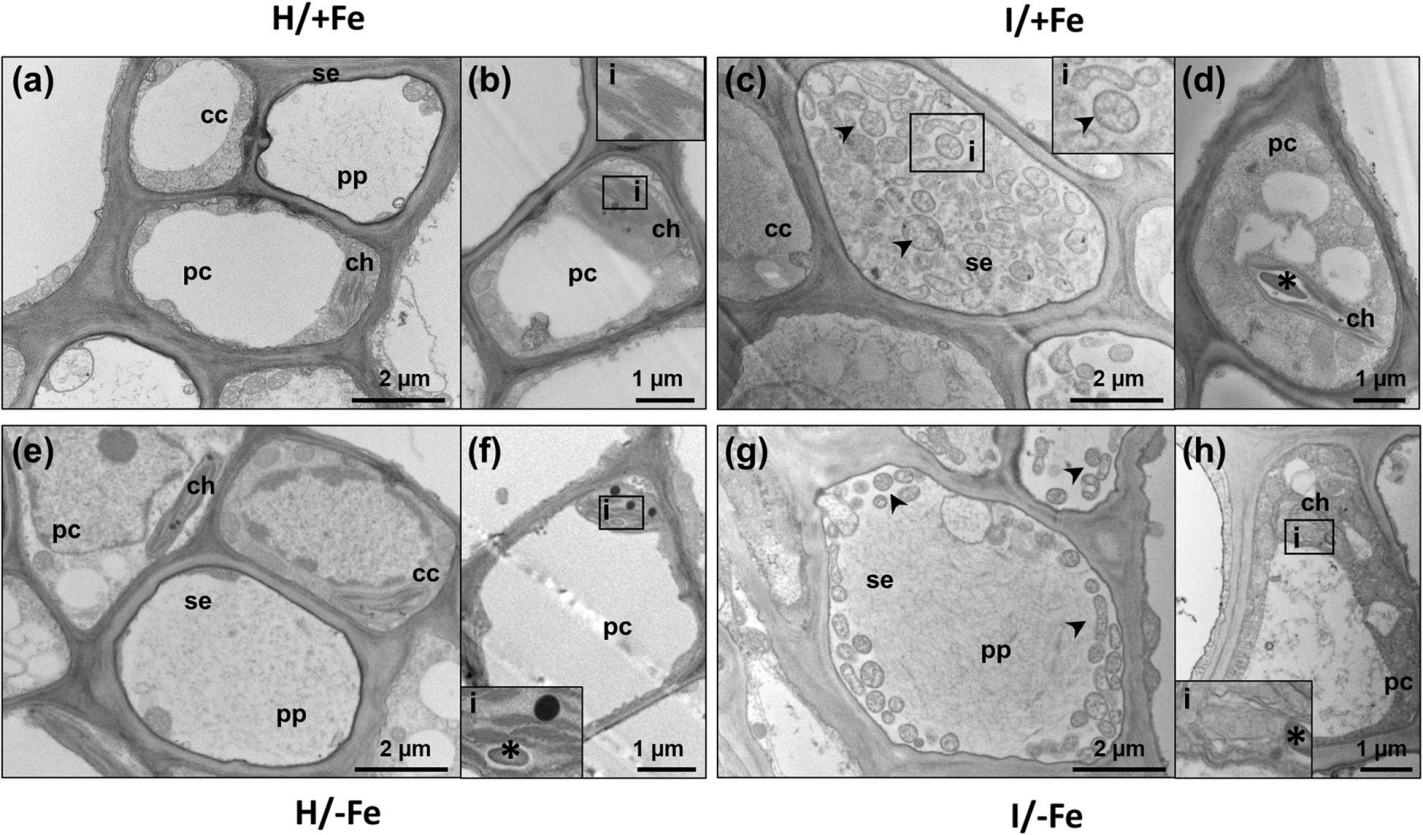
Effects of Fe starvation and phytoplasma infection on phloem ultrastructure. **a**-**d** Micrographs of healthy (H/+Fe) plants (**a**, **b**) and infected (I/+Fe) plants (**c**, **d**); phytoplasmas were detected exclusively in the lumen of the sieve elements (**c**). In companion and mesophyll cells, chloroplasts showed distorted arrangement of thylakoid stacks and significative accumulation of starch (**d**). **e**-**g** Micrographs of healthy Fe-starved (H/−Fe) tissues (**e**, **f**); sieve elements exhibited a regular ultrastructure (**e**), companion and parenchyma cells exhibited misshaped chloroplasts with large starch grains embedded between granal and stromal lamellae (**f**). **g**, **h** Phytoplasma-infected/Fe-starved (I/−Fe) plants with phytoplasma in sieve elements (**g**) and severely altered chloroplast ultrastructure (**h**). cc: companion cell; ch: chloroplast; i: inset; pc: parenchyma cell; se: sieve element; *: starch; arrowheads indicate phytoplasmas. Three non-serial cross sections from five plants were analysed for each condition (*n* = 15)

To visualize potential changes in cellular ultrastructure following pathogen infection or Fe starvation, leaf midribs were examined by TEM (Fig. 3). In samples from H/+Fe plants, TEM images revealed well-structured cells, tiny protein filaments were visible in the lumen of sieve elements (Fig. 3a). The chloroplasts in companion and phloem parenchyma cells were large and oval shaped, and contained fully developed grana with numerous layers and well-developed stroma lamellae (Fig. 3b). In I/+Fe plants, phytoplasmas with their typical pleomorphic profile were detected exclusively in the lumen of the sieve elements, surrounded by a pronounced accumulation of protein filaments (Fig. 3c). In companion and phloem parenchyma cells, chloroplasts showed irregular arrangements of thylakoid stacks, associated with large starch grains causing a distortion of the parallel pattern of the lamellae (Fig. 3d). Fe starvation did not alter sieve element ultrastructure (Fig. 3e), but affected thylakoid organization in companion and phloem parenchyma cells. Similar to infected plants, Fe-deficient plants showed disorganized grana and stroma lamellae and accumulation of starch (Fig. 3f). In I/−Fe tissues, similar to I/+Fe tissues, phytoplasmas were exclusively detected in sieve elements, plugged by massive depositions of phloem protein filaments (Fig. 3g). Chloroplasts were disorganized and exhibited severely altered ultrastructure (Fig. 3h).

### Iron distribution is altered by phytoplasma infection

**Fig. 4:**
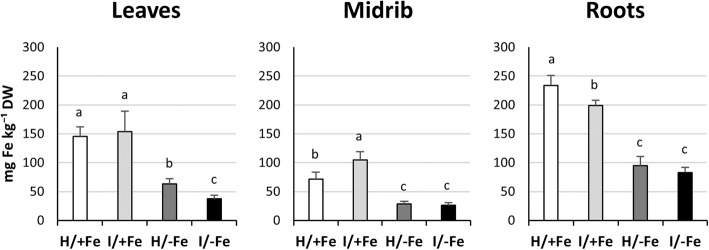
Effects of phytoplasma infection and Fe starvation on Fe concentration in whole leaves, midribs and roots. Fe concentration in whole leaves, leaf midribs and roots of H/+Fe, I/+Fe, H/−Fe, and I/−Fe tomato plants. Fe concentration was determined by ICP-OES. Results are expressed as mean ± SD (*n* = 6). DW: dry weight. Different letters indicate statistically significant differences (*P* < 0.05) among conditions (one-way ANOVA followed by Holm-Sidak’s test)

To investigate a possible effect of the infection on Fe uptake and translocation, the Fe content of leaves and roots was quantified by ICP-OES. Whereas in leaves of I/+Fe plants the Fe concentration was similar to that of H/+Fe plants, a significant decrease was observed in H/−Fe and I/−Fe plants (Fig. 4). Following Fe starvation, the Fe concentration decreased by 57% in H/−Fe plants and by 75% in I/−Fe plants relative to untreated plants. To focus on the Fe content of the infection region, leaf midribs were analysed in addition (Fig. 4). Surprisingly, I/+Fe midribs showed an Fe content that was 47% higher than that of healthy plants, while no difference was found between H/−Fe and I/−Fe midribs. In roots, I/+Fe plants exhibited a reduction of the Fe concentration by 15% in comparison to H/+Fe plants. As expected, Fe starvation caused a strong decrease in Fe concentration in roots of both H/−Fe and I/−Fe plants (60 and 65%, respectively, compared to H/+Fe, Fig. 4).

**Fig. 5:**
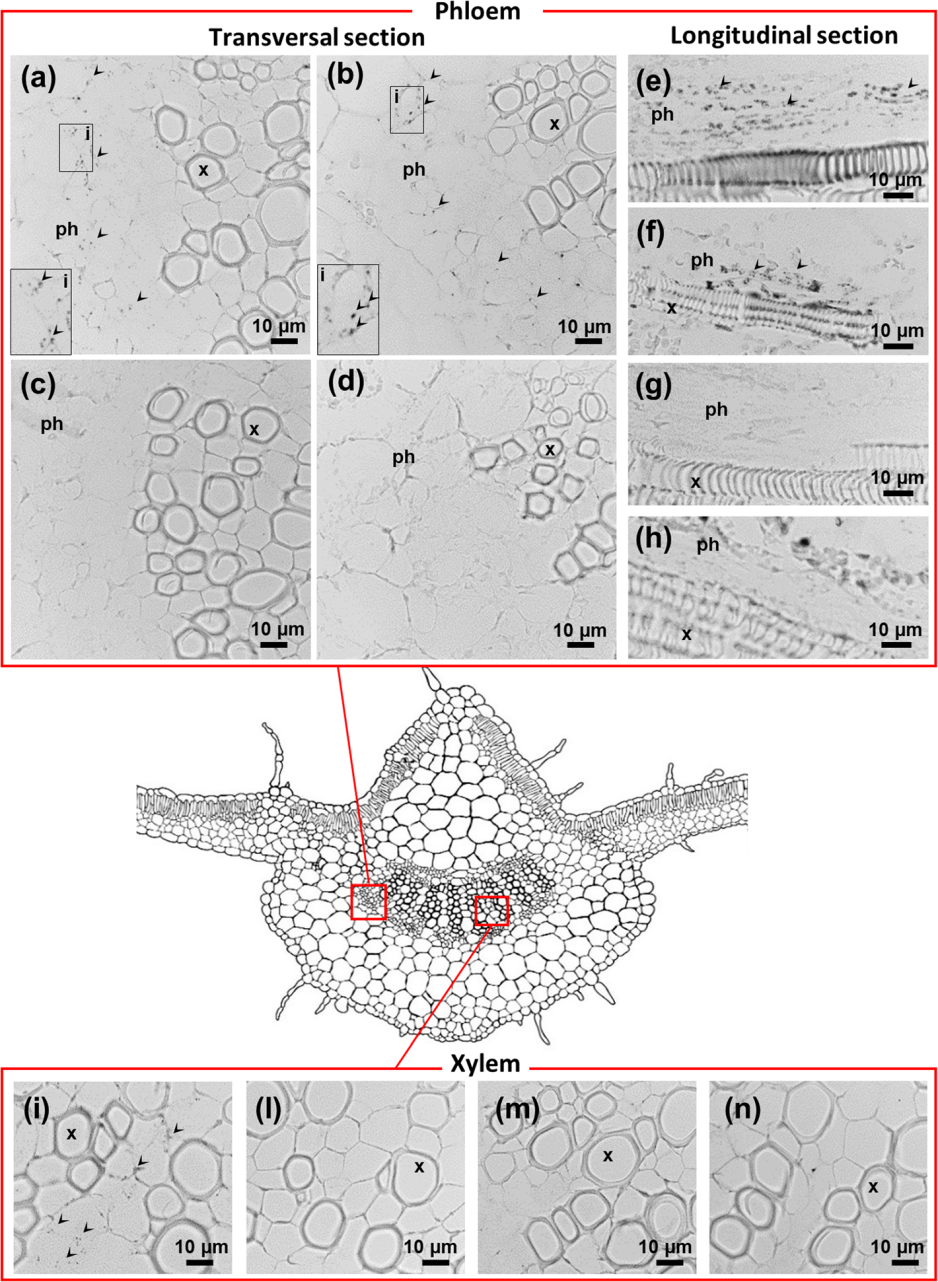
Effects of phytoplasma infection and Fe starvation on Fe distribution in the leaf midrib and surrounding parenchyma. **a**-**d** Perls’-DAB staining on 7 μm-thick transversal sections of leaf tissues in the phloem area of healthy (H/+Fe) plants (**a**), phytoplasma-infected (I/+Fe) plants (**b**), Fe-starved (H/−Fe) plants (**c**), and phytoplasma-infected/Fe-starved (I/−Fe) plants (**d**). **e**-**h** Longitudinal sections. Fe dots (arrow heads) are visualized in phloem cells of healthy and phytoplasma-infected/Fe-sufficient plants. **i**-**n** transversal sections of the xylem area. Fe dots are visualised only in H/+Fe plants. ph: phloem; x: xylem; arrowheads indicate Fe dots. Scale bars: 10 μm

Next, we investigated whether the presence of pathogens altered the distribution of Fe in leaves using Perls’-DAB staining. H/+Fe plants showed pronounced Fe staining in the phloem area (Fig. 5a; Additional file 1: Figure S1a), clearly visible in the longitudinal sections (Fig. 5e). Tiny Fe dots were also present in xylem parenchyma cells (Fig. 5i). Fe dots in the phloem area were also observed in midribs of I/+Fe plants (Fig. 5b, f; Additional file 1: Figure S1b). However, no Fe deposits in xylem parenchyma cells were found in infected plants (Fig. 5l). Thus, the increased Fe content of the infected midribs can be predominantly attributed to a higher Fe concentration in the phloem. Independent of their health status, in Fe-deficient plants Fe dots were neither detected in midrib cells nor in xylem or phloem tissue (Fig. 5c, d, g, h, m, n; Additional file 1: Figure S1c, d).

### Fe starvation and phytoplasma infection induce specific, partially overlapping changes in the transcriptome of tomato leaves

To gain insights into the transcriptional response to phytoplasma infection or Fe deficiency, single-end stranded RNA-seq transcriptome profiling was performed on midrib-enriched leaves. Phytoplasmas are phloem-restricted pathogens, thus midrib-enriched samples are usually analyzed to avoid an excessive dilution of transcripts. Here, to compare the two stresses we limited transcriptional profiling to control (H/+Fe), infected Fe-sufficient (I/+Fe), and Fe-deficient (H/−Fe) plants. After quality filtering, approximately 38 million reads for each of the nine libraries (three conditions, three biological replicates) were mapped to the reference genome on average, corresponding to a mean mapping rate of 83.1 ± 1.2%. On average, 20,463 ± 90 genes were detected in midrib-enriched leaves as being expressed with FPKM > 1 in at least one condition of each pairwise comparison. DEGs were defined by a FDR < 0.05 and FPKM > 1 in at least one condition.

**Fig. 6:**
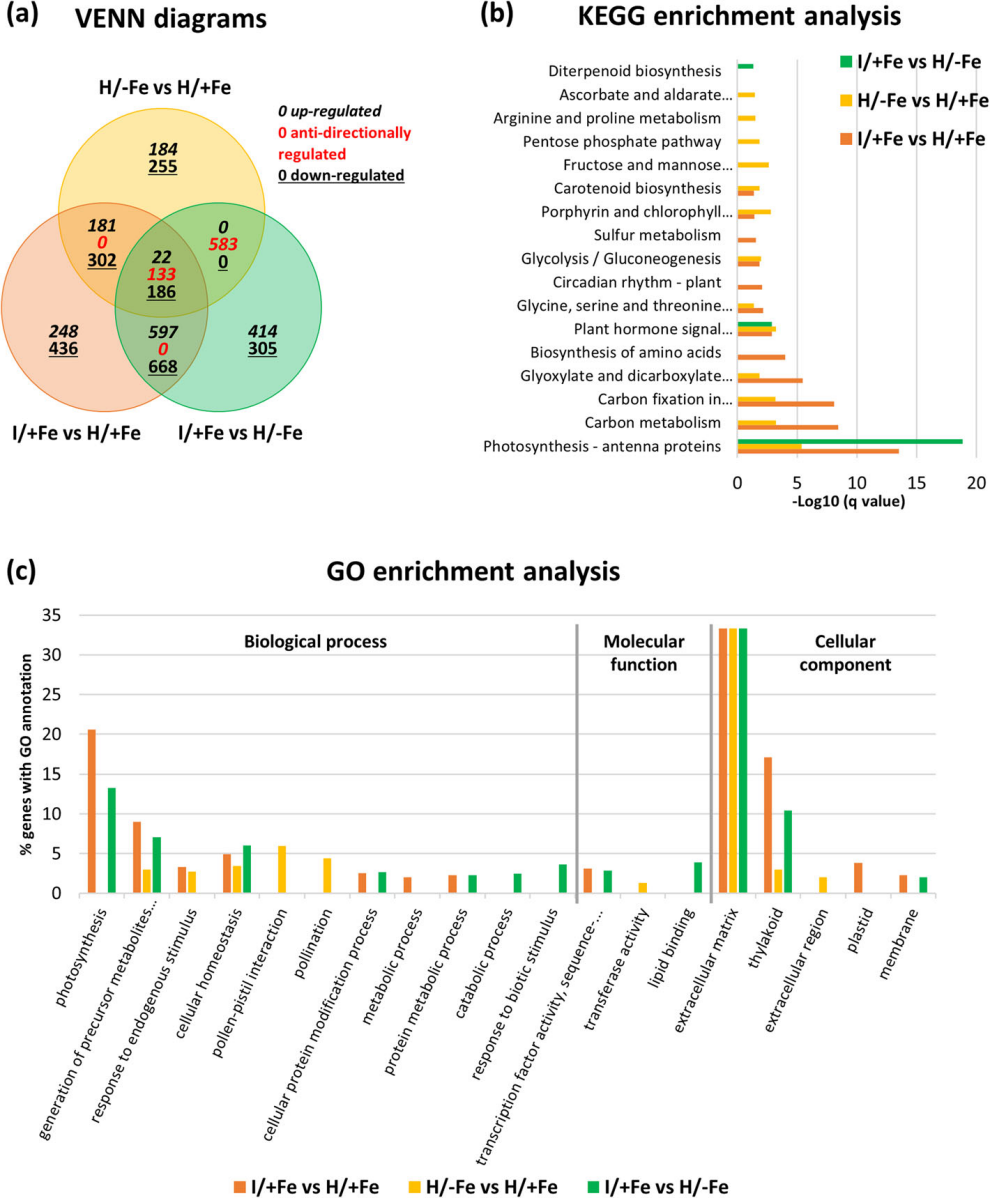
Venn diagram, KEGG pathway, and gene ontology (GO) enrichment analyses of differentially expressed genes (DEGs) in the three comparison groups. **a** Venn diagram showing the number of up-, down-, and anti-directionally regulated differentially expressed genes (DEGs) that were common and specific for the pairwise comparisons. **b** KEGG pathway enrichment. X-axis indicates the value of -Log10 (q value). **c** GO enrichment. The y-axis indicates the percentage of significant DEGs corresponding to the total number of genes annotated in each GO category (*P* < 0.05). DEGs were grouped into three major functional categories: biological process, cellular component, and molecular function

In Fe-sufficient plants, phytoplasma infection altered the expression of 2773 genes relative to controls (Fig. 6a: I/+Fe vs H/+Fe, orange circle). A subset of 1846 genes was classified as differentially expressed between H/−Fe and H/+Fe (Fig. 6a: H/−Fe vs H/+Fe, yellow circle). Comparing infected and Fe-deficient plants (Fig. 6a: I/+Fe vs H/−Fe, green circle) yielded 2908 DEGs. Among the subset of 341 genes common to all comparisons, only 89 of 133 DEGs were anti-directionally regulated by phytoplasma-infection and Fe-starvation, suggesting generally similar effects of phytoplasma infection and Fe deficiency on commonly targeted genes. Quantitative RT-PCR confirmed expression directionality and showed similar levels of regulation for all genes examined, indicating that fold-change values obtained from RNA-seq were accurate (Additional file 3: Table S3).

**Fig. 7:**
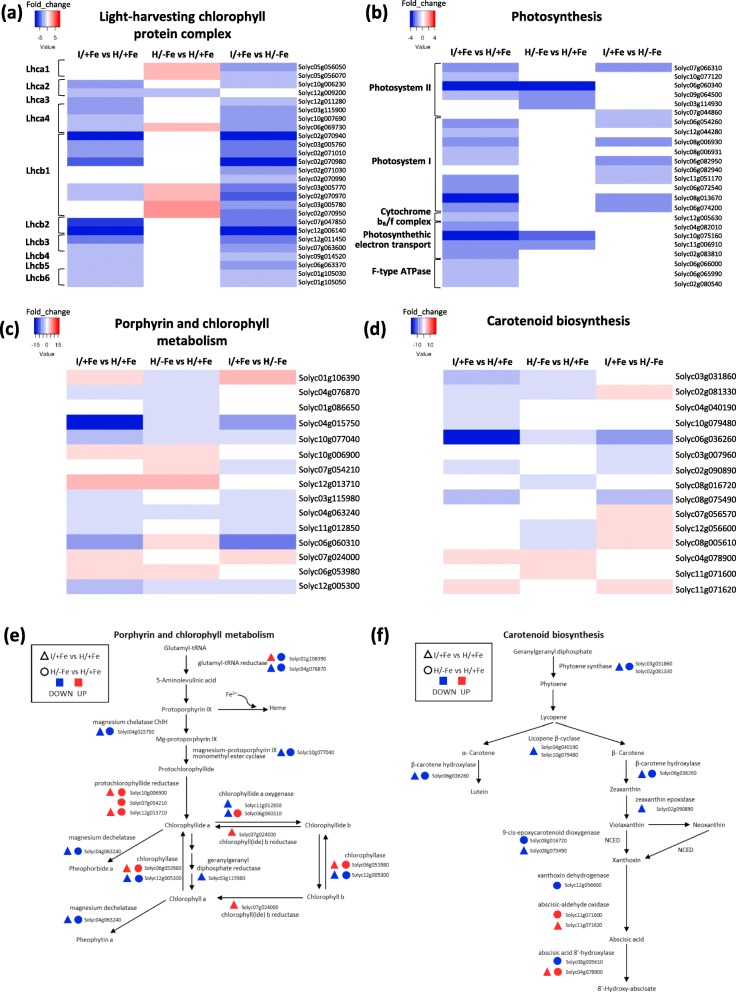
Heat map analysis of differentially expressed genes. **a**-**d** Fold changes of DEGs in the three comparison groups involved in antenna protein cluster (KEGG sly00196; **a**), photosynthesis-light reactions (sly00195; **b**), porphyrin and chlorophyll metabolism (sly00860; **c**), and carotenoid biosynthesis (sly00906; **d**). **e**, **f** Partial representation of porphyrin and chlorophyll (**e**) and carotenoid biosynthesis (**f**) pathways

The GO categories enriched in I/+Fe samples (i.e. photosynthesis, generation of precursor metabolites and energy, cellular homeostasis, thylakoid, and plastid; Fig. 6c) indicated that photosynthesis-related processes represent the major changes caused by the infection, which was confirmed by the most enriched KEGG pathways (Fig. 6b). Interesting, the most enriched group of phytoplasma-infected genes was the antenna protein cluster (KEGG pathway sly00196; Fig. 6b; Fig. 7a; Additional file 3: Table S4). Following infection, a general downregulation of several genes encoding antenna proteins associated with photosystem I (clustered in the orthologs group Lhca) and photosystem II (Lhcb group) was observed. Most DEGs were specific to this condition. In H/−Fe plants, several enriched GO terms were in common with I/+Fe plants (Fig. 6c), as well as most enriched KEGG pathways (Fig. 6b). Similar to what has been observed in I/+Fe plants, the enrichment analysis suggests that light harvesting and photosynthetic light reactions are major targets of Fe deficiency. Moreover, under both conditions KEGG enrichment listed several DEGs involved in carbon metabolism, specifically in carbon fixation and in the C2 cycle (Fig. 6b). When examining genes associated with porphyrin and chlorophyll metabolism (KEGG sly00860), genes encoding proteins involved in chlorophyll biosynthesis such as the glutamyl-tRNA reductase 1 (Solyc04g076870 and Solyc01g106390), the magnesium chelatase subunit H (ChlH; Solyc04g015750), and the putative magnesium-protoporphyrin monomethyl ester cyclase (at103; Solyc10g077040) were found to be down-regulated upon Fe deficiency, indicating that, as expected, chlorophyll biosynthesis was negatively affected by the Fe regime (Fig. 7c, e; Additional file 3: Table S5). A similar trend was observed in I/+Fe plants. However, as observed for a subset of genes encoding LHC proteins, some genes involved in porphyrin metabolism were anti-directionally regulated by phytoplasma infection and Fe starvation. For example, one of the two genes encoding glutamyl-tRNA reductase (Solyc01g106390), which represents a key step for the biosynthesis of both heme and chlorophyll, was 2-fold induced in I/+Fe leaves but downregulated in Fe-deficient plants. Also, a chlorophyllide a oxygenase gene (CAO; Solyc06g060310) was down-regulated in I/+Fe plants, while up-regulated in H/−Fe. In order to avoid the accumulation of unquenched chlorophyll molecules and subsequent generation of ROS, changes in the abundance of LHC apoproteins are generally accompanied by parallel changes in chlorophyll content. All genes involved in chlorophyll turnover where affected by both stresses. Also carotenoid biosynthesis was affected in both conditions. Here, a similar trend in gene regulation in response to both stresses was observed, i.e. downregulation of the genes encoding key enzymes involved in the biosynthesis of alpha- and beta-carotene and their oxidized forms from geranylgeranyl bisphosphate through phytoene and lycopene intermediates. Noteworthy, also the cleavage of violaxanthin, a C40 precursor that is critical for abscisic acid biosynthesis, was downregulated in response to both stresses (Fig. 7d, f; Additional file 3: Table S6).

Beside the light harvesting apparatus, which appeared to be compromised in all components, i.e. antenna proteins synthesis and pigment biosynthesis, several clusters of genes associated with photosynthetic light reactions (KEGG sly00195) were also down-regulated under both conditions. Common targets of both stressors were the two ferredoxin genes (PetF; Solyc10g075160, Solyc11g006910), and two genes associated with photosystem II (PsbS and Psb28). In addition, phytoplasma infection targeted genes of the electron transport chain (i.e. the plastocianin encoding gene Solyc04g082010 and a ferredoxin-NADP^+^ reductase gene; Solyc02g083810), and several genes encoding proteins of the other thylakoid complexes, i.e. the photosystem I, the cytochrome *b*_*6*_*/f* complex, and the F-type ATP synthase complex (subunit gamma and b) (Fig. 7b; Additional file 3: Table S7).

### Transcriptional profiling reveals robust regulation of genes involved in flowering time, transport, and photosynthesis in infected and Fe-deficient plants

To identify genes that are regulated by Fe starvation or phytoplasma infection, and possibly play key roles in the plant responses to these cues, we considered the top 100 (on fold-change basis) up- or downregulated genes in plants subjected to either stress condition (total FPKM expression level > 10). In leaves of Fe-deficient plants, two putative Arabidopsis *IRON MAN* (*IMA*) orthologs (Solyc12g006770, Solyc12g00675) were most strongly induced (Table 1, Additional file 4: Table S8). IMA is a family of Fe deficiency-induced peptides that has been associated with the communication of the Fe status from leaves to roots via the phloem, recently identified in Arabidopsis [65]. Several other genes putatively encoding IMA peptides were also robustly induced upon Fe deficiency but were not expressed in leaves of Fe-sufficient plants. Similarly, *bHLH68* (Solyc10g079680), an ortholog of *AtbHLH38/39*, was induced upon Fe deficiency and not expressed under control conditions. A gene encoding a putative ortholog of OLIGOPEPTIDE TRANSPORTER 3 (OPT3), a phloem-specific, plasma membrane protein that has been implicated in long-distance signalling in Arabidopsis (Solyc11g012700) [73–75], was also upregulated upon Fe starvation. A gene encoding the Fe sequestration protein ferritin (Solyc06g050980) was downregulated under Fe-deficient conditions. The expression of the putative tomato *NEET* ortholog (Solyc03g007030), a protein with a conserved role in Fe metabolism reactive oxygen homeostasis, decreased in response to Fe starvation, a response that has also been observed in Arabidopsis leaves [76]. In addition, three genes encoding proteins with similarity to vacuolar Fe transporters of the VIT family (Solyc04g071165, Solyc04g051180, Solyc01g104780) were among this subset, indicating reduced sequestration of Fe under Fe-deficient conditions. Also, several genes involved in the transport of mineral nutrients such as Pi and boron showed reduced expression in Fe-deficient leaves. A massive downregulation upon Fe deficiency was observed for RuBisCO activase 1 (*RCA1*; Solyc09g011080), suggesting strongly reduced photosynthetic activity in Fe-deficient plants. Reduced expression of a group of genes encoding proteins involved in flowering control such as EARLY FLOWERING 4 (Solyc06g051680) and three CONSTANS-LIKE proteins (Solyc07g045180, Solyc07g045185, Solyc02g093590) is indicative of delayed flowering of Fe-deficient plants.

**Table 1:** Genes involved in flowering time, transport, photosynthesis and defence in Fe-deficient and infected plants

SGN locus	NCBI Gene ID	NCBI description / 1st blastp hit / 2nd blastp hit	Fold-change	Total FPKM
-Fe/H vs + Fe/H			Upregulated	
Solyc12g006770		#N/D	75.2	405.5
Solyc12g006750		#N/D	nan	271.1
Solyc11g012700	101265194	Oligopeptide transporter 3	4.8	207.9
Solyc10g079680	101258211	Putative transcription factor SlbHLH068	nan	33.1
-Fe/H vs + Fe/H			Downregulated	
Solyc06g050980	104647958	Ferritin-1, chloroplastic	7.0	306.6
Solyc09g011080	101250725	Ribulose bisphosphate carboxylase/oxygenase activase 1, chloroplastic	7.4	192.4
Solyc01g102610	101246763	Ferric reduction oxidase 6	5.5	168.1
Solyc01g079150	101260863	Boron transporter 1	4.2	65.7
Solyc04g071165	101264000	Ref|XP_004237778.1| PREDICTED: vacuolar iron transporter homolog 1-like [*S.l*.]	6.8	25.1
Solyc02g088240	101247877	Ref|XP_004232204.1| PREDICTED: phosphate transporter PHO1 homolog 3 [*S.l*.]	9.2	22.6
Solyc07g045185	101267825	Ref|XP_004243599.1| PREDICTED: zinc finger protein CONSTANS-LIKE 10 [*S.l*.]	10.0	22.1
Solyc07g045180	101267825	Ref|XP_004243599.1| PREDICTED: zinc finger protein CONSTANS-LIKE 10 [*S.l*.]	11.4	19.6
Solyc06g051680		Ref|XP_009629950.1| PREDICTED: protein EARLY FLOWERING 4-like [*N.t.*]	5.3	19.2
Solyc04g051180		Ref|XP_015072233.1| PREDICTED: vacuolar iron transporter homolog 1-like [*S.p*.]	5.8	15.5
Solyc03g007030	101244055	CDGSH iron-sulfur domain-containing protein	7.6	15.5
Solyc09g082550	101253320	Sulfate transporter 3.1-like	9.2	12.9
Solyc01g104780	101246768	Vacuolar iron transporter homolog 4-like	11.3	10.8
Solyc02g093590	101256821	Zinc finger protein CONSTANS-LIKE 16	5.7	10.0
I/+Fe vs H/+Fe			Upregulated	
Solyc06g051680		Ref|XP_009629950.1| PREDICTED: protein EARLY FLOWERING 4-like [*N.t*.]	4.5	89.11
Solyc11g066130	543971	osmotin-like protein	5.2	18.8
Solyc07g007760	101263826	defensin-like protein	4.9	1534.2
Solyc02g082920	544149	chitinase, CHI3	3.1	1365.6
Solyc10g055810	544148	chitinase, CHI9	2.8	453.7
Solyc02g082930	544147	chitinase, CHI17	1.7	103.1
Solyc10g055800	101267358	endochitinase 4	3.0	289.2
Solyc05g050130	101253788	acidic endochitinase	2.6	305.4
Solyc00g174340	544123	pathogenesis-related leaf protein 6, PR1B1	2.1	4507.3
Solyc09g007010	544185	pathogenesis-related protein P4	1.9	152.3
Solyc08g080650	544082	osmotin-like protein OSML13	2.4	719.1
I/+Fe vs H/+Fe			Downregulated	
Solyc10g086580		Ref|XP_010312360.1| ribulose bisphosphate carboxylase/oxygenase activase, chloroplastic LOC101249777	9.0	3523.3
Solyc06g054270	101261239	Sugar transport protein 8-like	9.9	297.4
Solyc09g090570	101262255	Protein PROTON GRADIENT REGULATION 5, chloroplastic	18.8	229.3
Solyc06g073180	778334	CONSTANS interacting protein 1	16.3	220.1
Solyc09g011080	101250725	Ribulose bisphosphate carboxylase/oxygenase activase 1, chloroplastic	209.3	170.3
Solyc01g080870	101250924	Protein NRT1/ PTR FAMILY 7.3	13.3	152.0
Solyc01g102610	101246763	Ref|XP_004230384.1| PREDICTED: ferric reduction oxidase 6-like [*S.l.*]	42.4	145.5
Solyc02g089540	778253	CONSTANS 1;CO1;ortholog	66.2	60.2
Solyc01g079150	101260863	Ref|XP_004229368.1| PREDICTED: boron transporter 1 [*S.l.*]	69.4	53.7
Solyc07g053140	101265452	Ref|XP_004243424.1| PREDICTED: zinc finger protein CONSTANS-LIKE 4-like [*S.l.*]	33.9	38.4
Solyc08g077170	101263538	Ref|XP_004245877.1| PREDICTED: protein NRT1/ PTR FAMILY 7.3 [*S.l.]*	42.5	19.9
Solyc12g005660	101055534	Hop-interacting protein THI121	13.1	19.9
Solyc05g010060	101244953	Phosphate transporter PHO1 homolog 1	10.8	13.3
Solyc04g050440	544110	Ammonium transporter	19.1	13.2
Solyc05g024260	101255592	Bidirectional sugar transporter N3	20.1	12.4
Solyc09g082550	101253320	Ref|XP_004247591.1| PREDICTED: sulfate transporter 3.1-like [*S.l.*]	41.5	11.9
Solyc04g072740	101245940	Low affinity sulfate transporter 3	10.2	10.8

Fold-change is the ratio of gene expression level (FPKM) in the indicated pairwise comparison. Total FPKM corresponds to the sum of expression level of the corresponding gene in the two conditions. *Nan* not a number. See Additional file 4: Table S8 for further details

Similar to Fe-deficient plants, the expression of *RCA1* was repressed as a response to phytoplasma infection. In diseased plants, also a second *RCA* gene (Solyc10g086580) was downregulated. In addition, the gene encoding PROTON GRADIENT REGULATION 5 (Solyc09g090570), a protein required for cyclic electron transport and preventing of oxidative damage to photosystem I [77] showed reduced activity in infected plants. Associated with a supposedly reduced photosynthetic rate, *SUGAR TRANSPORT PROTEIN 8-LIKE* (*STP11,* Solyc06g054270) and a bidirectional sugar transporter belonging to the SWEET family (Solyc05g024260) were downregulated upon pathogen infection. Similar to Fe-deficient plants, several genes putatively related to flowering (Solyc06g073180; Solyc02g089540; Solyc07g053140; Solyc12g005660) showed reduced expression in diseased plants. Further, for a suite of genes encoding proteins involved in the transport of boron (Solyc01g079150), phosphate (Solyc05g010060), sulphate (Solyc04g072740; Solyc09g082550), ammonium (Solyc04g050440), nitrate (Solyc08g077170), potassium (Solyc07g014680), and a ferric reductase (FRO6, Solyc01g102610), reduced transcript abundance was observed, indicating a generally reduced translocation of mineral nutrients in diseased plants. It is worthy of note that, consistent with the high Fe content of the infected phloem, the expression of both *IMA* and *OPT3* orthologs was not changed. Upregulated in infected plants were several proteins related to pathogen defence, the pathogenesis-related thaumatin superfamily protein Solyc11g066130, and *DEFENSIN-LIKE PROTEIN 3* (Solyc07g007760). Several other pathogen defence-related genes were expressed at high levels and significantly but only moderately upregulated and were thus not included in the list of the top 100 upregulated genes (Table 1). Among these genes were several chitinases (*CHI3*, Solyc02g082920; *CHI9*, Solyc10g055810; *CHI17*, Solyc02g082930; *ENDOCHITINASE 4*, Solyc10g055800; *ACIDIC ENDOCHITINASE* Solyc05g050130), and genes encoding pathogenesis-related proteins such as pathogenesis-related leaf protein 6 (PR1b1, Solyc00g174340) and pathogenesis-related protein P4 (P4/pr1a, Solyc09g007010). In addition, other thaumatin-like proteins such as osmotin-like protein *OSML13* (TPM-1, Solyc08g080650) were among these moderately induced genes.

### Phytoplasma infection perturbs the Fe deficiency response of tomato roots

**Fig. 8:**
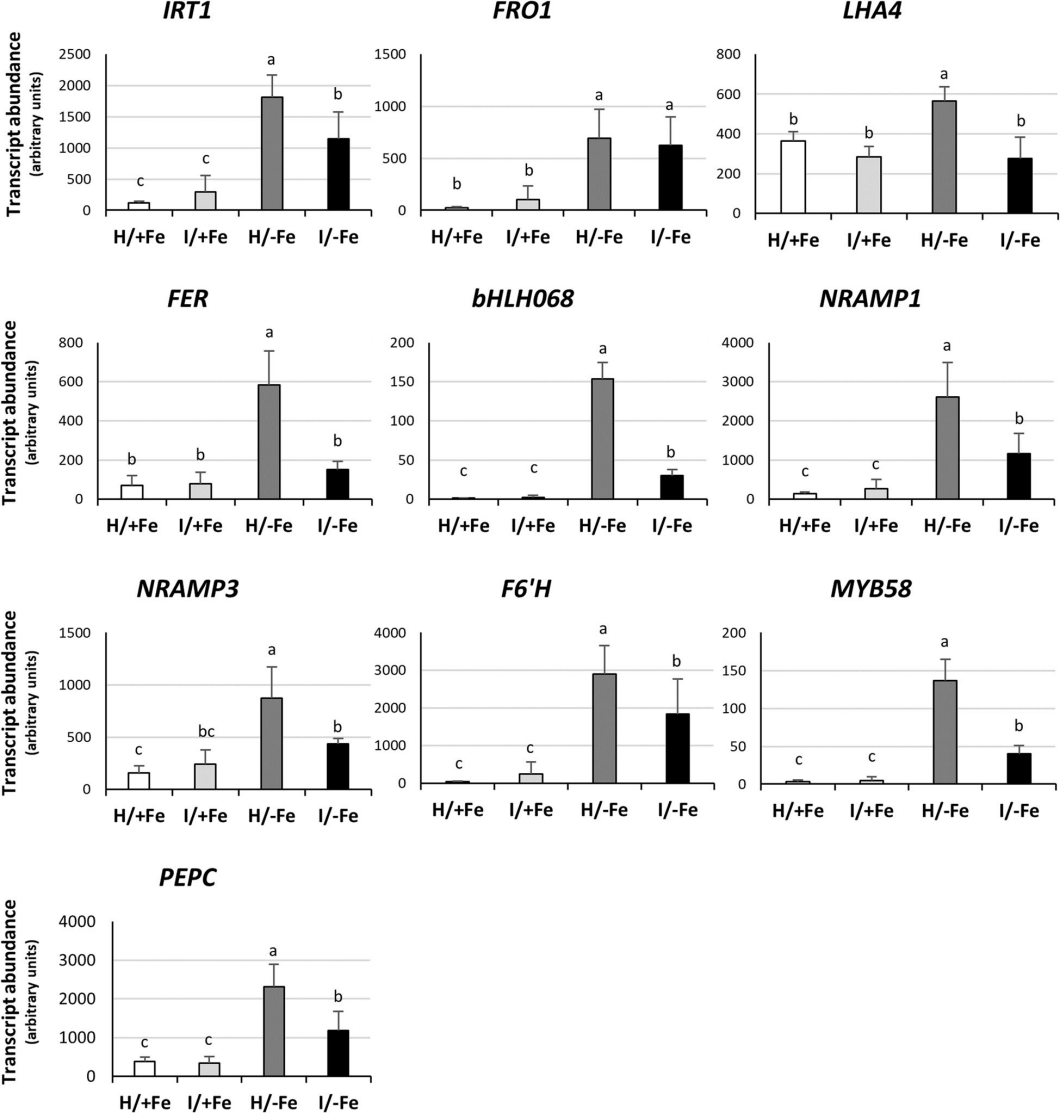
Expression analysis of Fe-related genes in tomato roots by RT-qPCR. The mean normalized expression (MNE) of each gene is plotted as the transcript abundance compared with the *UPL3* expression level (set at 100). Results are expressed as mean ± SD (*n* = 5). Different letters indicate statistically significant differences (*P* < 0.05) among the conditions (one-way ANOVA followed by Holm-Sidak’s test)

To investigate if phytoplasma infection affects the Fe acquisition mechanism at the root level, the expression of the *IRON-REGULATED TRANSPORTER 1* (*IRT1;* Solyc02g069200) [44], the *FERRIC REDUCTION OXIDASE 1* (*FRO1;* Solyc07g017780) [78] and the *AtAHA2* ortholog *LHA4* (Solyc07g017780) [79] was analysed by RT-qPCR (Fig. 8). Expression analysis involved also two transcription factors known to act upstream in the regulation of Fe uptake genes, the *AtFIT* ortholog *FER* (Solyc06g051550) [42], and *SlbHLH068* (Solyc10g079680), which interacts with FER to regulate the Fe deficiency response in tomato [60] (Fig. 8). In addition, we quantified the transcripts level of other genes known to be involved in intra-cellular metal transport and mobilization of metal pools, i.e. *NRAMP1* (Solyc11g018530) and *NRAMP3* (Solyc02g092800; Fig. 8) [80]. The tomato genome database was further explored for genes possibly involved in the synthesis and activation of Fe-mobilizing coumarins [see [52] for a review]. *SlF6’H1* (Solyc11g045520), which is annotated as feruloyl CoA *ortho*-hydroxylase 1 in the NCBI gene database, shares 63% identity at the amino acid level with the scopoletin 8-hydroxylase AtS8H (AT3G12900), a protein involved in the biosynthesis of fraxetin [50, 51, 81]. Arabidopsis *MYB72* is a root-specific transcription factor functioning as a node of convergence in the induced systemic resistance and Fe starvation signalling pathways, triggering the activation of coumarins via β-glucosidase BGLU42 [66, 82]. In the Hierarchical Catalog of Orthologs (OrthoDB; https://www.orthodb.org), the *Solanum lycopersicum* ortholog of AtMYB72 is MYB DOMAIN PROTEIN 58 (SlMYB58*;* Solyc10g005550), which possesses a homeobox domain-like, a Myb, and a SANT/Myb domain (InterPro domains IPR009057, 017930, and 001005, respectively) similar to AtMYB72. Finally, we analysed the expression of a phospho*enol*pyruvate carboxylase (*PEPC*) gene. PEPC is involved in CO_2_ fixation and subsequent synthesis of organic acids, especially citrate, that transport Fe to leaves via the xylem, and contribute with other organic molecules to the mobilization of Fe from the apoplast in roots [39, 83]. SlPEPC (Solyc10g007290) aligns with the highest score and 88% identity to Arabidopsis PPC3 (AT3G14940), the PEPC isoenzyme that is most abundantly expressed in Arabidopsis roots.

In the presence of Fe, the expression level of the genes under investigation was not significantly modified by phytoplasma infection, although high variability among individuals has potentially masked possible differences between infected and non-infected plants (Fig. 8). As expected, all investigated gene were up-regulated upon Fe deficiency, although the degree of the induction greatly varied among genes. For instance, *bHLH068* was induced by a factor of 139, while *LHA4* was increased only 1.5-fold. When examining I/−Fe plants, an increase in the expression of most of the genes under investigation was observed, but, unexpectedly, the extent of induction was lower than that of H/−Fe plants (Fig. 8). Thus, the general Fe deficiency-induced upregulation that characterized both healthy and infected plants differed in a significant manner according to the sanitary status of the plants, as for almost all of the investigated genes transcript abundance was reduced by the presence of phytoplasma. The containment of the upregulation varied according to the gene considered, ranging from a decrease in expression from 36.8% (*IRT1*) to 80% (*bHLH068*). A notable exception to this trend was the expression of *FRO1*, which was induced by growth on Fe-free media regardless of the sanitary status. This result was confirmed by the Fe^3+^-chelate reduction activity survey that was performed on excised roots (Additional file 2: Figure S2). In accordance with the gene expression analysis of *FRO1*, reductase activity was induced by Fe deficiency but remained unaffected by phytoplasma infection.

## Discussion

### The transcriptional response of phytoplasma-infected tomato leaves mirrors Fe deficiency

Fe appears to play a central role in the interaction between pathogens and their plant hosts. In the current study, both phytoplasma-infected and Fe-starved plants developed chlorotic leaves and displayed a concomitant decrease in total chlorophyll content, as indirectly indicated by SPAD values. Moreover, as previously reported, both Fe deficiency and phytoplasma infection altered the ultrastructure of chloroplasts, causing disorganization of thylakoids [22, 28, 84–86]. Both stresses compromised photosystem II, the soluble component of the electron transport chain, and LHCs by modulating the expression of several antenna genes and impairing key steps in the biosynthesis of chlorophyll and carotenoids. In order to avoid photo-oxidative damage, the carotenoid biosynthetic pathway is linked to the biosynthesis of chlorophyll and the expression of chlorophyll-binding proteins. The inhibition of the expression of genes involved in photosynthesis is in accordance with previously reported results of plant-phytoplasma interaction studies [23–29], an observation that is possibly linked to altered Fe distribution caused by the infection. In the present study, most genes encoding proteins involved in photosynthetic light reactions, porphyrin / chlorophyll metabolism, and in carotenoid biosynthesis had comparable expression changes in both I/+Fe and H/−Fe plants. This pattern suggests that plants have evolved control mechanisms to avoid deleterious reactions of light absorption when the photosynthetic activity is impaired. Several components of the photosynthetic apparatus were modulated in a partly overlapping manner in Fe-deficient and infected plants. As regards antenna proteins, LHC-encoding genes were upregulated in Fe-deficient plants, the same genes were dramatically downregulated in infected plants, suggesting different cause-effect scenarios under pathogen infection and Fe deficiency. However, modulation of genes activity was observed in *Malus halliana* seedlings, where Fe deficiency induced a short-term downregulation of several genes involved in photosynthesis antenna proteins followed by upregulation of the same subset of genes and subsequent recovery of photosynthesis after a three-day Fe deficiency [87]. However, a subset of genes was oppositely regulated by infection and Fe starvation, suggesting the induction of at least disparate signalling cascades. Noteworthy is the downregulation of genes encoding components of the photosystem I, the cytochrome *b*_*6*_*/f* complex, the F-type ATPase, and lchb proteins, only in I/+Fe plants. Chloroplasts accommodate many biosynthetic pathways, including those for hormones, and produce ROS, which readily interact with components of the phytohormone-signalling network to regulate defence pathways [88]. It is intriguing to observe that such a disruption of chloroplast function could further advantage the phytoplasma, as shown in other pathosystems [89].

### Phytoplasma infection alters the local distribution of Fe

Both phytoplasma infection and Fe starvation caused a decrease in chlorophyll content and chlorosis, and induced similar alteration in the transcriptome regarding photosynthesis, and chlorophyll and carotenoid metabolism. Considering that different to other pathogens, phytoplasmas are strictly restricted to phloem tissues, it appears reasonable to assume that phytoplasma infection alters the spatial distribution of Fe due to the locally restricted demand of the pathogen. The combination of Perls’-DAB staining and Fe quantification focused on leaf midribs confirmed this supposition, suggesting the occurrence of a spatial shift of Fe from the surrounding tissues to the infection site. This phenomenon has been observed in other plant-pathogen systems such as Arabidopsis infected by *Dickeya dadantii*. Here, a loss of Fe from cellular compartments and the cell wall was associated with the concomitant accumulation of Fe inside and around the bacteria [90]. It further appears that Fe starvation imposed on infected plants reduced the phytoplasma titre, corroborating the assumption that phytoplasmas must acquire Fe from the phloem, converting the phloem into a sink tissue for Fe.

### Phytoplasma infection perturbs the Fe deficiency response in roots

**Fig. 9:**
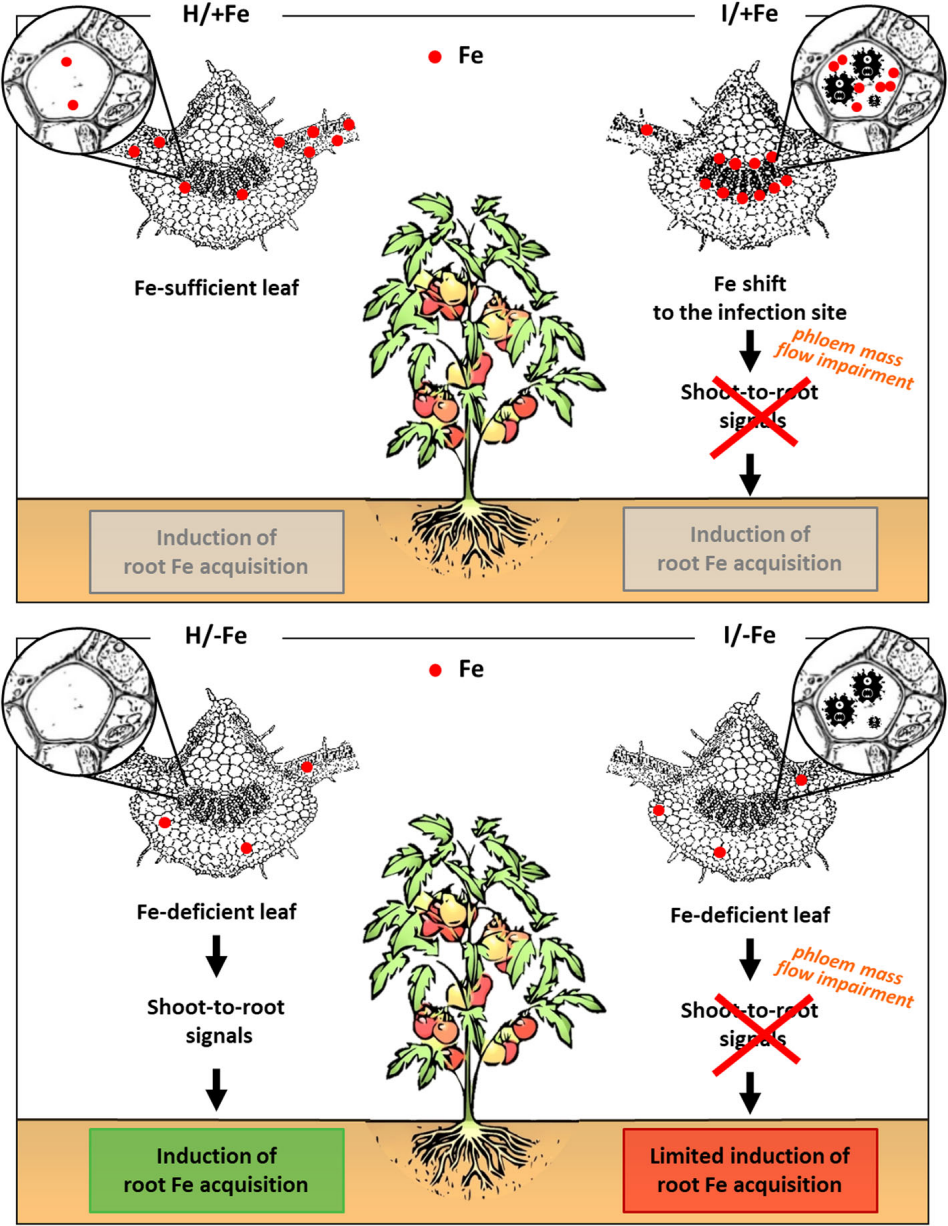
Model summarizing the effect of phytoplasma infection and/or Fe deficiency on Fe content and Fe acquisition. In healthy tomato plants grown on Fe-sufficient conditions (H/+Fe), Fe is distributed in the whole leaf. In Fe-sufficient, infected leaf (I/+Fe), Fe content is increased in the midrib, concentrating in the phloem tissue. This shift towards the infection site does not induce any root response. Independent of their health status, Fe-deficient plants H/−Fe and I/−Fe) show an extremely reduced Fe content, with no peculiar distribution. In these conditions, the Fe acquisition mechanism is induced, but less induced when the phloem is infected. An impairment of the phloem mass flow and/or an interference with signals moving in the phloem are suggested for phytoplasma infection

Several lines of evidence support the assumption that shoots can signal their Fe status to the roots, therefore tuning Fe uptake from the soil [91]. In healthy plants, Fe starvation led to a considerable upregulation of the Fe uptake genes in roots. The same genes were also induced in infected Fe-starved plants, although to a lower extent than healthy Fe-starved plants. The decreased expression of Fe uptake genes in infected Fe-deficient plants may be caused by the interference of phytoplasma with the transport of a promotive long-distance signal in the phloem that modulates root Fe uptake. Such an interference is not perceived in Fe-sufficient conditions, where the local changes in Fe distribution appear to have relatively minor effects on the overall Fe metabolism of the host, although the lower Fe concentration detected in roots seems to suggest it. This presumptive restriction of shoot-to-root signalling is in line with the phloem mass flow impairment by phytoplasma infection demonstrated in vivo [17, 92]. A recent work had identified a novel family of peptides (IMA) expressed preferentially in the phloem in the regulation of Fe responses in roots by acting upstream of the master transcription factor FIT [65]. The transport of IMA peptides could be altered in infected plants. Split-root experiments showed that the expression of *IRT1* and *FRO2* is controlled by both local and systemic signalling pathways and that both signals are integrated to tightly control the production of the root iron uptake proteins [93–96]. Notably, in our system *FRO1* seemed to respond chiefly to a local signal. A model is proposed showing the phytoplasma interference with the Fe distribution in leaf and the transport of a promotive long-distance signal moving in the phloem (Fig. 9).

## Conclusions

In conclusion, it appears that phytoplasmas must acquire Fe from the phloem, converting the phloem into a sink tissue for Fe. We found that photosynthesis and porphyrin synthesis are the main targets of phytoplasma infection and Fe starvation, leading to the development of chlorotic leaves, and, presumably, reduced photosynthetic rates. Additive effects of the two stressors on chlorosis and chlorophyll content support the idea of parallel but separate routes towards the phenotypic readouts. While Fe deficiency directly affects chlorophyll synthesis, in infected plants chlorosis and impaired photosynthesis rather seem to be related to impaired signalling and subsequent deregulation of the genes involved in these processes. Under Fe-deficient conditions, the presence of phytoplasmas may compromise the communication of the Fe status between leaves and roots, possibly by interference with the synthesis or transport of a promotive signal. Moreover, restricted source-sink transport of various classes of compounds such as carbohydrates and hormones may cause short circuits and negatively feedback on metabolic and physiologic processes of the leaves.

## Methods

### Plant material and growth conditions

Tomato (*Solanum lycopersicum* L., cv. Micro-Tom) seeds were kindly provided by Dr. Sabrina Palmano (CNR_IPSP, Torino, Italy). Seeds were collected from fruits of one single plant and germinated for 7 days in the dark at 22 °C between two layers of filter paper soaked in 1 mM CaSO_4_. Homogenous seedlings were transferred into hydroponic nutrient solution containing 1.5 mM K_2_SO_4_, 3 mM KNO_3_, 0.5 mM MgSO_4_, 1.5 mM CaCl_2_, 0.5 mM NaH_2_PO_4,_ 25 μM H_3_BO_3_, 1 μM MnSO_4_, 0.5 μM ZnSO_4_, 0.3 μM CuSO_4_, 0.05 μM (NH_4_)_6_Mo_7_O_24,_ and 20 μM Fe-EDTA. The pH was adjusted to 6.0 with KOH. The aerated nutrient solution was replaced every 4 days. Plants were grown in a greenhouse at 20–25 °C with a 16 h light photoperiod. After 4 weeks, half of the plants were infected with ‘*Ca. P. solani*’, belonging to the stolbur subgroup 16SrXII-A [97], by grafting shoot tips from phytoplasma-infected tomato plants onto healthy tomato plants. Healthy shoot tips were grafted onto the remaining half of the plants. Two weeks after grafting, Fe starvation was induced in one half of the healthy plants and one half of the infected plants by growing plants in Fe-free nutrient solution during the last three weeks of the experiment. All plant samples were collected 5 weeks after grafting. Plants were grown in four different conditions: healthy or phytoplasma-infected plants grown with full nutrient solution containing Fe (H/+Fe and I/+Fe, respectively), and healthy or phytoplasma-infected plants grown during the last 3 weeks in Fe-free nutrient solution (H/−Fe and I/−Fe, respectively). For transcriptome profiling by RNA-seq, we focused the analysis on three conditions: H/+Fe, I/+Fe, and H/−Fe.

### Plant biometrics and phytoplasma detection

Biometric analyses were performed on six plants per condition. Total plant fresh weight was recorded at the end of the experimental period. Chlorophyll was indirectly determined by measuring leaf light transmittance with a portable chlorophyll meter (SPAD-502; Minolta, Osaka, Japan). For each plant, five SPAD measurements were taken on five leaves. Average leaf area was determined by analysing five leaves per plant. Leaf area was calculated using the ImageJ 1.49 m software package (National Institutes of Health, Bethesda, MD, USA).

The presence of phytoplasma was assessed in healthy and symptomatic plants by qPCR analysis. Total genomic DNA was extracted from approximately 800 mg of leaf tissue enriched in midribs according to Doyle & Doyle [98] modified by Martini et al. [99]. DNA concentration and purity were verified using a NanoDrop 1000 spectrophotometer (Thermo Fisher Scientific, Wilmington, DE, USA). Phytoplasma detection was carried out using specific primers designed on the 16SrRNA gene of ‘*Ca. P. solani*’ (GenBank accession no. AF248959) according to Santi et al. [30].

### Phytoplasma relative quantification

Phytoplasma titre was determined in eight plants per condition (I/+Fe and I/−Fe) by qPCR analysis of ‘*Ca. P. solani*’ and relative quantification of specific DNA levels. Total genomic DNA was extracted as described above. In each experiment, duplicate samples were amplified in a qPCR reaction targeting the 16SrRNA gene of ‘Ca. *P. solani*’ and the single-copy tomato gene nicotianamine synthase (*Chloronerva*, *CHLN)* [100] as internal positive reference. The primers for *16SrRNA* were the same than those used for phytoplasma detection (see above). The primers for *CHLN* are listed in Additional file 3: Table S2. For each gene, qPCR analysis was performed in triplicates in a 15 μL reaction mix, containing SsoAdvanced Universal SYBR Green Supermix (Bio-Rad Laboratories), 400 nM of primers for *16SrRNA* or 300 nM of primers for *CHLN*, and 2 μL template DNA normalised to 5 ng/μL. The reactions were performed as described in Santi et al. [19]. A positive and a negative control were run on every plate. To correct for inter-plate variation, a calibrator sample was run on every plate, allowing manual adjustment of the threshold level in order to maintain the threshold cycle (Ct) values of the calibrator sample constant. For each sample, phytoplasma DNA was determined by normalizing 16SrRNA gene relative to the tomato *CHLN*.

### Transmission electron microscopy

To preserve phloem tissue structures, a specifically adapted protocol was used to prepare samples for transmission electron microscopy (TEM) observation, as reported for tomato [21]. Thirty mm long midrib segments were excised from three leaves of five plants per experimental condition. Ultrathin sections (60–70 nm in thickness) were stained with UAR-EMS uranyl acetate replacement stain (Electron Microscopy Sciences, Fort Washington, PA, USA), and observed under a PHILIPS CM 10 (FEI, Eindhoven, The Netherlands) TEM operated at 80 kV, equipped with a Megaview G3 CCD camera (EMSIS GmbH, Münster, Germany). Three non-serial cross sections from each sample were analysed.

### RNA-seq

Single-end stranded RNA-seq transcriptome analysis was performed on tomato leaves. Two leaves from three plants each were pooled and considered as one biological replicate. Three biological replicates for each of three conditions (H/+Fe, I/+Fe and H/−Fe) were analysed. In total, nine libraries were prepared as follows. Circa 1 g of leaf tissue enriched in midribs was ground in liquid nitrogen, and total RNA was extracted from approximately 100 mg of powder with the Spectrum Plant Total RNA Kit (Sigma-Aldrich, St Louis, MO, USA) according to the manufacturer’s instructions. DNA was removed using the TURBO DNA-free™ Kit (Life Technologies, Carlsbad, CA, USA). The quality of RNA was evaluated using a Bioanalyzer 2100 (Agilent Technologies, Santa Clara, CA, USA). RIN scores ranged from 6.0 to 7.8. Libraries were prepared from 200 ng of total RNA with the TruSeq stranded Total RNA library Prep Plant Kit (Illumina Inc., San Diego, CA, USA) following the manufacturer’s instructions. This kit enables bead-based depletion of ribosomal RNA in multiple plant species. Libraries were sequenced on the Illumina NextSeq500 platform as 75 bp single-end stranded reads. Quality analysis of RNA, library construction, and RNA-seq were carried out at IGA Technology Services (Udine, Italy), who provided adapter-trimmed sequences and raw reads in Fastq-files. For each library, more than 45 millions of reads were obtained.

### RNA-seq data analysis

Read quality was analysed by FastQC (www.bioinformatics.babraham.ac.uk/projects/fastqc). The first six bases, which showed anomalous enrichments, were trimmed by FASTX_trimmer, and reads with a quality score below 30 (for more than 50% of the bases) were removed by FASTX quality filter application (http://hannonlab.cshl.edu/fastx_toolkit). Clean reads were mapped to the reference genome of the cultivar Heinz 1706, Build SL3.0 and gene annotation ITAG3.20 (release date: June 15, 2017; https://solgenomics.net/organism/Solanum_lycopersicum/genome) by TopHat 2.0.9 [101]. Default parameters were used except for segment mismatch that was set to no more than 1, minimum intron length to 25 bp, and maximum intron length to 200,000. Anchor length was set to 8, and maximum number of mismatches that can appear on the anchor region was set to zero. Differentially expressed genes (DEGs) were identified by Cuffdiff 2.1.1 [102] using multiple-hit correction, min-alignment-count 10, normalization to known transcripts, and a False Discovery Rate (FDR) set to 0.05. Visualization of read densities from RNA-seq was performed using the Integrated Genome Browser [103]. The DEGs among the comparisons were graphically represented by Venn diagram entering the DEGs identifiers in VennPlex [104]. Quality control, trimming, RNA-seq alignment and quantification were performed on CyVerse cyberinfrastructure (www.cyverse.org).

For functional annotation of sequences and data mining, the PANTHER (Protein Analysis Through Evolutionary Relationships) classification system was used to classify genes and their proteins in families, subfamilies, and molecular function. NCBI Entrez was used to retrieve further functional annotation. Further information on genes for which no annotation was available was retrieved by aligning all the protein sequences available in the tomato annotation against the NCBI database with the Blastp software (restricted to viridiplantae to reduce computation time), considering matches with an e-value lower than 10^-9. Gene ontology classifications (GO) of DEGs in the three comparisons were downloaded from Sol Genomics Network FTP site for the ITAG3.20 annotation release. The enrichment for the differential GO term distribution in DEGs was tested by Fisher’s exact test, implemented in the R package topGO. Metabolic pathway analysis was performed using the KEGGenrich function in the R package clusterProfiler [105]. The KEGG database was used for functional characterization of genes and their organization in metabolic pathways [106]. *P* value cut-off for significance of enrichment tests was set to 0.05.

### ICP-OES analysis

Fe concentration was measured by Inductively Coupled Plasma–Optical Emission Spectroscopy (ICP-OES) analysis in both leaves (whole leaf or midrib) and roots, in six plants for each condition. Root apoplastic Fe pools were removed as described by Bienfait et al. 1985 [107]. Root and leaf tissues were dried at 65 °C for 48 h, then at 105 °C for 24 h. Dried samples (200 mg) were then suspended in 10 ml of concentrated HNO_3_ [65% (v/v)] in Teflon vessels, and digested in a microwave oven (CEM Mars Xpress Matthews, NC, USA), according to the USEPA 3052 method “Plant Xpress” (USEPA, 1995). The microwave temperature was increased to 180 °C for 10 min at 1600 W (ramp time 30 min). Samples were than diluted to 20 ml with ultrapure deionized water and filtered with 0.45 μm PTFE filters. Elemental concentration was subsequently determined by ICP-OES (Varian Vista Pro axial) after dilution of the samples [8.8 ml of ultrapure deionized water, 0.2 ml Yttrium (Y) standard solution 50 mg L^− 1^ as internal standard, and 1 ml of filtered sample]. Mineral quantifications were carried out using a certified multi-element standard. Tomato leaves (NIST SRM 1573a) were used as external certified reference material. Mineral nutrient concentration in leaves was expressed on a dry weight (DW) basis.

### Perls’-DAB staining

For in situ Perls’-DAB Fe staining intensification, leaves were fixed in a solution containing 2% (w/v) paraformaldehyde, 1% (v/v) glutaraldehyde, 1% (w/v) caffeine, and 0.01% triton X-100 in 0.1 M phosphate buffer (pH 7) for 24 h. Fixed tissue was dehydrated in 10, 30, 50, 60, 70, 80, 90, and 100% ethanol for 1 h at each concentration and then embedded in paraffin. Sections (7 μm) were obtained using a microtome (Leica, Milan, Italy), placed on poly-l-lysine-coated slides (Menzel-Glaser, Braunschweig, Germany), and dried at 30 °C for 1 h. Before staining, sections were dewaxed and rehydrated. Leaves sections were incubated for 45 min in 4% (v/v) HCl and 4% w/v K-ferrocyanide (Perls stain solution) for 45 min [73], except for negative controls which were incubated in 4% (v/v) HCl. After washing with deionized water, glass slides were incubated in a methanol solution containing 0.01 M NaN_3_ and 0.3% (v/v) H_2_O_2_ for 1 h, and then washed with 0.1 M phosphate buffer (pH 7.4). For the intensification reaction, samples were then incubated between 10 and 30 min in a 0.1 M phosphate buffer (pH 7.4) solution containing 0.025% (w/v) DAB (Sigma), 0.005% (v/v) H_2_O_2_, and 0.005% (w/v) CoCl_2_ (intensification solution) [108]. Rinsing with distilled water stopped the reaction. Samples were observed by a light microscope (Nikon Eclipse Ni microscope, Tokyo, Japan).

### Quantitative RT-PCR

To investigate gene expression, RT-qPCR experiments were performed on a CFX96 instrument (Bio-Rad Laboratories, Richmond, CA, USA). About 1 g of root tissue for each plant was homogenized by mortar and liquid nitrogen, and RNA was extracted from approximately 100 mg of powder with the Spectrum Plant Total RNA Kit (Sigma-Aldrich, St Louis, MO, USA) according to the manufacturer’s instructions. Extracted RNA was DNase-treated and reverse-transcribed into complementary DNA (cDNA) with the QuantiTect Reverse Transcription Kit (Qiagen GmbH, Hilden, Germany) following the manufacturer’s instructions. To identify the most suitable reference gene, gene stability measures (M values) were calculated on different genes according to the geNorm program [109] (Additional file 3: Table S1). The *UPL3* gene was found to be stably expressed genes in both leaves and roots (M = 0.303 and M = 0.357, respectively). Primer pair efficiency (E) was evaluated as described in [110] using standard curves of different dilutions of pooled cDNA. SsoFast EvaGreen Supermix 2x (Bio-Rad Laboratories Inc., Hercules, CA, USA) and cDNA obtained from 2.5 ng of RNA, and specific primers (final concentration 300 nM of each primers) were used in a total volume of 15 μL for all genes analysed. Under these conditions, E of all primer pairs was =2. Reaction was performed as described in [21]. Gene and primer sequences for expression analysis are reported in Additional file 3: Table S2. Mean normalized expression (MNE) for each gene of interest [111] was calculated by normalizing its mean expression level to the level of the *UPL3* gene. Three technical repeats and five individuals were used for MNE determination.

To validate the data obtained by RNA-seq, expression patterns of selected DEGs were analysed by RT-qPCR, using the same RNA that was used for library construction and sequencing. Primers were designed to the corresponding sequences retrieved from SGN (Additional file 3: Table S2).

### Fe^3+^-chelate reduction activity

Fe^3+^-chelate reduction (FCR) activity in roots was assayed by the method described in [112]. Briefly, lateral roots were excised from five plants per condition and embedded in a gel consisting of 0.2 mM CaSO_4_, 1% (w/v) agarose, 5 mM MES buffer (pH 5.5), 0.1 mM Fe^3+^-EDTA, and 0.3 mM Na_2_-bathophenanthrolinedisulfonic acid (BPDS). The reddish coloured staining, which is related to the reduction activity of Fe^3+^ to Fe^2+^ and the simultaneous Fe^2+^-BPDS complex formation, developed in 30 min.

### Statistical analysis

Data are expressed as mean values ±SD. Statistical analyses were performed by SigmaPlot 12.0 (SigmaPlot Software, CA, USA), using one-way ANOVA with a Holm-Sidak’s test as post hoc test for multiple comparisons.

## Additional files

Additional file 1: **Figure S1.** Fe detection in tomato leaf midribs. Perls’-DAB staining on 7 μm-thick sections of leaf midribs in healthy plants (a), infected plants (b), Fe-starved plants (c), and infected Fe-starved tomato plants (d). Small Fe dots are visible in H/+Fe and I/+Fe conditions in the phloem area (a, b). (e) Control sections with DAB without previous Perls reaction. ph: phloem; x: xylem; arrowheads indicate Fe dots. Scale bars: 100 μm. (PNG 1417 kb)

Additional file 2: **Figure S2.** Qualitative visualization of Fe^3+^ reduction activity along lateral tomato roots. Roots were placed in 1% agarose containing 0.2 mM CaSO_4_, 5 mM Mes buffer (pH 5.5), 0.1 mM Fe^3+^-EDTA and 0.3 mM BPDS. The reddish coloration, corresponding to Fe^2+^-BPDS complex, reveales the regions of Fe^3+^ reduction only in H/−Fe and I/−Fe roots. Gel shown is representative of five independent experiments. For each condition, H/+Fe, I/+Fe, H/−Fe, and I/−Fe, five plants were examined, using two lateral roots (*n* = 5). (PNG 1192 kb)

Additional file 3: **Table S1.** List of primers and accession number of sequences used for housekeeping gene individuation. **Table S2.** Gene and primer sequences for root expression analysis and RNA-seq validation. **Table S3.** Experimental validation of a subset of genes regulated by phytoplasma-infection or Fe-starvation. **Table S4.** Genes associated with Photosynthesis-Antenna Proteins KEGG pathway (00196) in all pairwise comparisons. **Table S5.** Genes associated with ‘Porphyrin and chlorophyll metabolism’ KEGG pathway (00860) in all pairwise comparisons. **Table S6.** Genes associated with Carotenoid Biosynthesis KEGG pathway (00906) in the pairwise comparisons. **Table S7.** Genes associated with Photosynthesis-light reactions KEGG pathway (00195) in all pairwise comparisons. (DOCX 55 kb)

Additional file 4: **Table S8.** Top 100 up- or downregulated regulated genes in all pairwise comparisons. (XLSX 71 kb)

## Data Availability

The RNA-seq data generated and analysed during the current study have been deposited at the NCBI Sequence Read Archive with the BioProject ID: PRJNA548138 (https://www.ncbi.nlm.nih.gov/bioproject/548138).

## Abbreviations

AHA: *Arabidopsis* H^+^-ATPase
bHLH: basic Helix-Loop-Helix transcription factor
BPDS: Na_2_-bathophenanthrolinedisulfonic acid
Ca. *P. solani*: *Candidatus* Phytoplasma solani’
CAO: Chlorophyllide a oxygenase
chl: chlorophyll
ChlH: Magnesium Chelatase subunit H
Chln: Chloronerva
DAB: DAB-4HCL / 3,3′-Diaminobenzidine tetrahydrochloride
DEGs: Differentially regulated genes
FDR: False Discovery Rate
FIT: FER-like Iron deficiency induced Transcription factor
FRO: Ferric reduction oxidase / Fe^3+^-chelate reductase
GO: Gene ontology
ICP-OES: Inductively Coupled Plasma-Optical Emission Spectroscopy
IMA: Iron man gene
IRT1: IRON-REGULATED TRANSPORTER 1
KEGG: Kyoto encyclopedia of genes and genomes
LHA: *Lycopersicum* H^+^-ATPase
LHC: Light-harvesting complex
OPT3: OLIGOPEPTIDE TRANSPORTER 3
PEPC: Phospho*enol*pyruvate carboxylase
PSI: Photosystem I
PSII: Photosystem II
qPCR: quantitative PCR
RCA1: RuBisCO activase 1
RNA-seq: RNA sequencing
RT-qPCR: quantitative reverse transcriptase-PCR
SlF6’H1: feruloyl CoA *ortho*-hydroxylase 1
TEM: Transmission Electron Microscopy
