# Supplementary figures and images for: ‘Candidatus Phytoplasma solani’ interferes with the distribution and uptake of iron in tomato
Source: BMC Genomics. 2019 Sep 10;20:703. doi: 10.1186/s12864-019-6062-x (PMC6734453; doi:10.1186/s12864-019-6062-x)

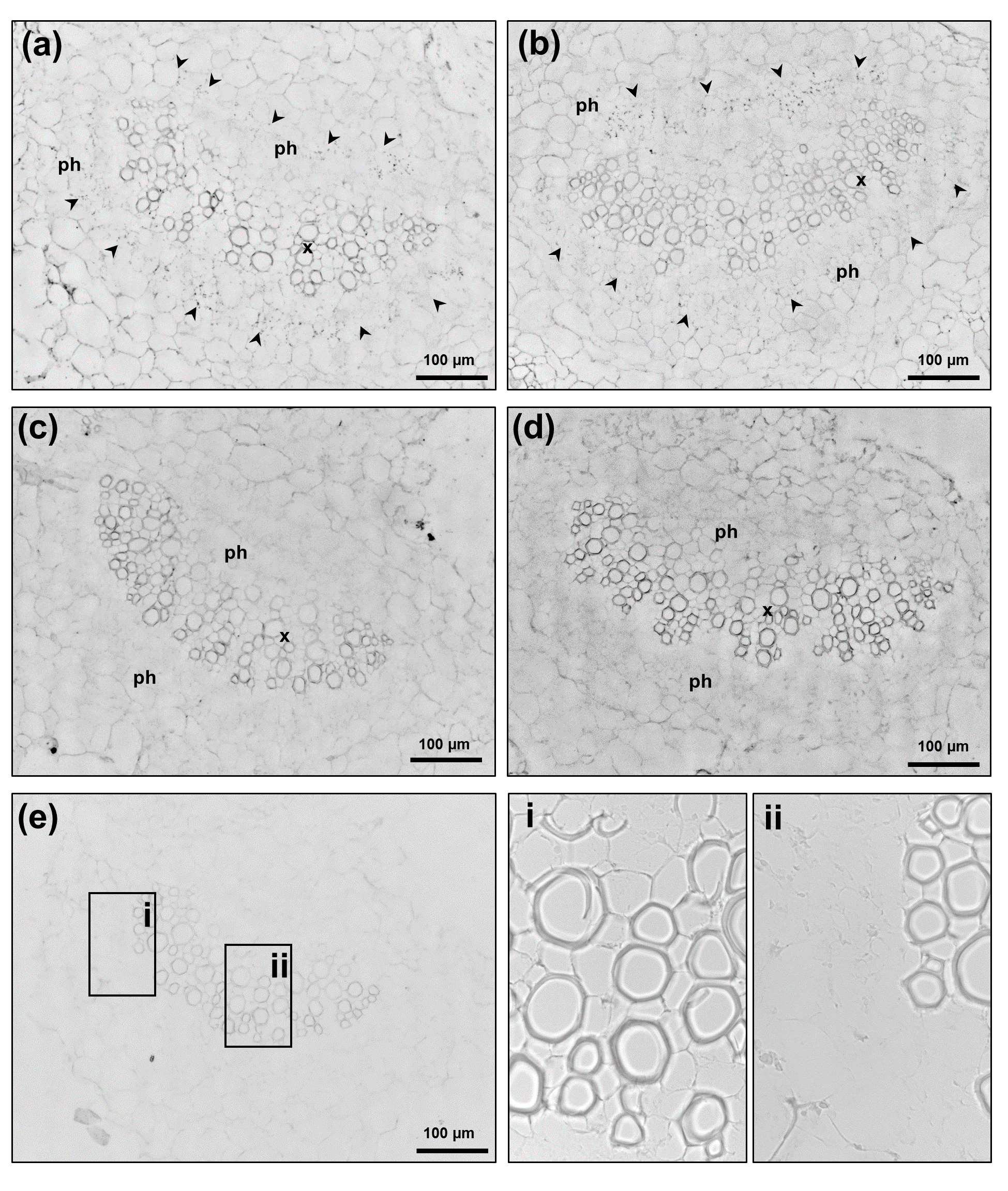

Supplement: Supplementary file 1 — Figure S1. Fe detection in tomato leaf midribs. Perls’-DAB staining on 7 μm-thick sections of leaf midribs in healthy plants (a), infected plants (b), Fe-starved plants (c), and infected Fe-starved tomato plants (d). Small Fe dots are visible in H/+Fe and I/+Fe conditions in the phloem area (a, b). (e) Control sections with DAB without previous Perls reaction. ph: phloem; x: xylem; arrowheads indicate Fe dots. Scale bars: 100 μm. (PNG 1417 kb) [file 12864_2019_6062_MOESM1_ESM.png]

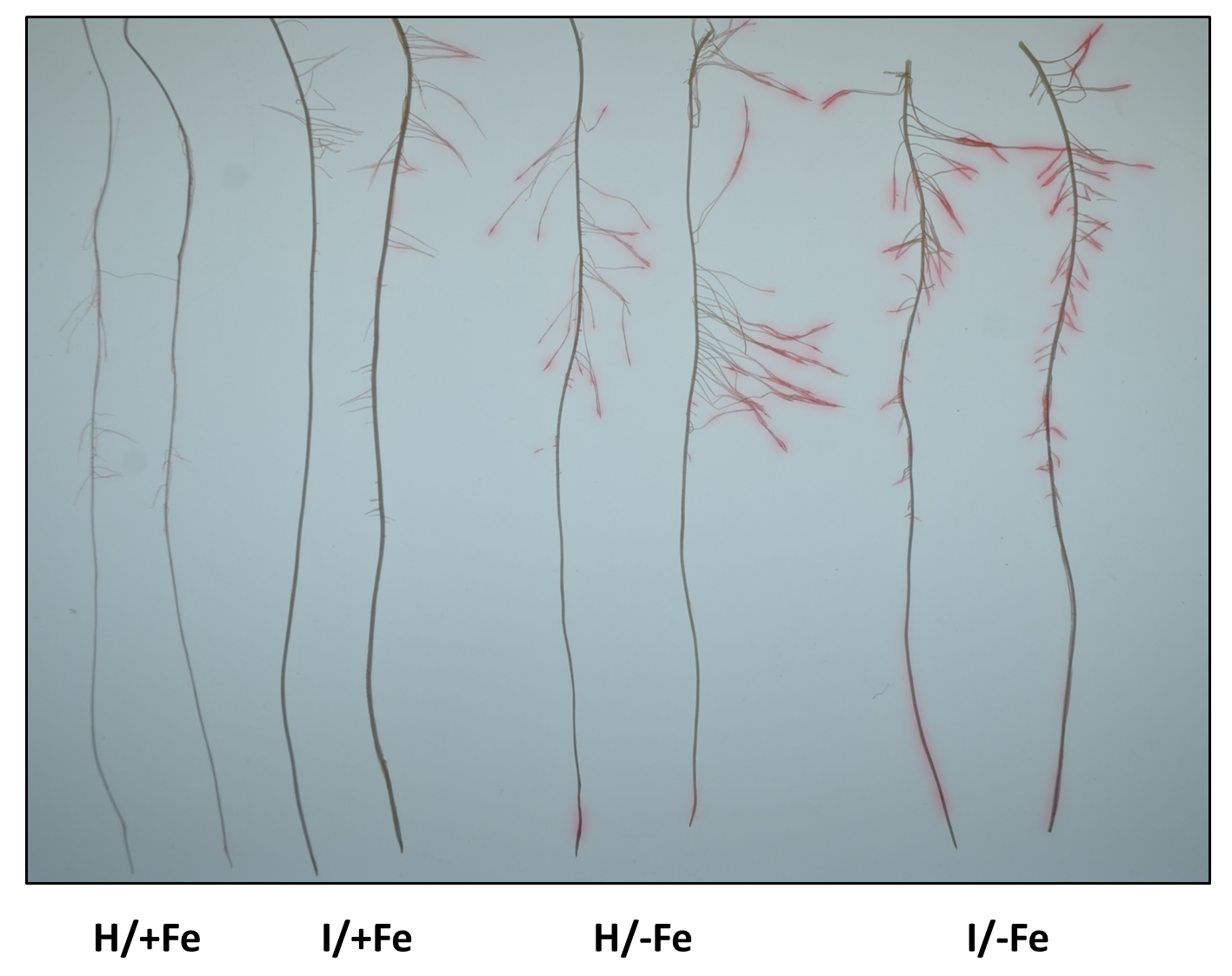

Supplement: Supplementary file 2 — Figure S2. Qualitative visualization of Fe3+ reduction activity along lateral tomato roots. Roots were placed in 1% agarose containing 0.2 mM CaSO4, 5 mM Mes buffer (pH 5.5), 0.1 mM Fe3+-EDTA and 0.3 mM BPDS. The reddish coloration, corresponding to Fe2+-BPDS complex, reveales the regions of Fe3+ reduction only in H/−Fe and I/−Fe roots. Gel shown is representative of five independent experiments. For each condition, H/+Fe, I/+Fe, H/−Fe, and I/−Fe, five plants were examined, using two lateral roots (n = 5). (PNG 1192 kb) [file 12864_2019_6062_MOESM2_ESM.png]
