# Supplementary material for: ‘Candidatus Phytoplasma solani’ interferes with the distribution and uptake of iron in tomato
Source: BMC Genomics. 2019 Sep 10;20:703. doi: 10.1186/s12864-019-6062-x (PMC6734453; doi:10.1186/s12864-019-6062-x)
Supplement: Supplementary file 3 — Table S1. List of primers and accession number of sequences used for housekeeping gene individuation. Table S2. Gene and primer sequences for root expression analysis and RNA-seq validation. Table S3. Experimental validation of a subset of genes regulated by phytoplasma-infection or Fe-starvation. Table S4. Genes associated with Photosynthesis-Antenna Proteins KEGG pathway (00196) in all pairwise comparisons. Table S5. Genes associated with ‘Porphyrin and chlorophyll metabolism’ KEGG pathway (00860) in all pairwise comparisons. Table S6. Genes associated with Carotenoid Biosynthesis KEGG pathway (00906) in the pairwise comparisons. Table S7. Genes associated with Photosynthesis-light reactions KEGG pathway (00195) in all pairwise comparisons. (DOCX 55 kb) [file 12864_2019_6062_MOESM3_ESM.docx]

**Table S1.** List of primers and accession number of sequences used for housekeeping gene individuation. *UPL3*: E3 ubiquitin-protein ligase UPL3; *EF-1*: elongation factor 1-alpha; *ACT-7 like:* actin-7-like; *TUB:* beta-tubulin.

| **Gene** | **Forward primer 5’-3’** | **Reverse primer 5’-3’** | **NCBI ID** | **SGN Gene ID** |
| --- | --- | --- | --- | --- |
| ***UPL3**** | TGTGAGGACTGGAATTGGGC | CAAGCGTCTCAGCCTTCCAT | XM_004230989.3  XM_010317077.2  101264868 | Solyc10g055450 |
| ***EF-1*** | GAGGCAAACTGTTGCTGTGG | TCCGTGCTCATCAAATGCA | XM_004240531.3  101244084 | Solyc06g009970 |
| ***ACT-7 like*** | TAGCACCTTCCAGCAGATGT | CAGCAGACCCGAGTTCACTT | NM_001321306.1  101262163 | Solyc11g005330 |
| ***TUB*** | TCCAAGTTTTGGTGACTTGAACC | ACAGCCAATTTCCTCAGGTCT | NM_001247878.2  778227 | Solyc04g081490 |

*This primer pair amplifies every gene transcript variant.

**Table S2.** Gene and primer sequences for root expression analysis and RNA-seq validation.

| **Gene** | **Forward primer 5’-3’** | **Reverse primer 5’-3’** | **NCBI Gene ID** | **SGN Gene ID** |
| --- | --- | --- | --- | --- |
| ***IRT1*** | GGGCTATCACTAGGTGCGTC | ATACTCCGCCTGTAGGATGC | 543597 | Solyc02g069200 |
| ***FER*** | CAAAGGGCGACACATTGCAG | TCTCTCACATAAAGAGTGAAGGTGA | 543705 | Solyc06g051550 |
| ***bHLH068*** | TGCAAGTGTAGAGGAAGATGGA | TCAATTGGTCCTTGCATCTGA | 101258211 | Solyc10g079680 |
| ***LHA4*** | GCTTTGATTTTCGTGACCCGT | TGGCAACCAATTGGGCAATCA | 101263827 | Solyc07g017780 |
| ***FRO1*** | AAGGGTGAAGGAAGTTGGTCC | ATCATGCCTTAGAAAATGTGTGGAA | 543871 | Solyc01g094910 |
| ***F6’H1*** | AGGAAATGGCTTTGGAATGGA | TCAAGAGCCACATCCTTGCAT | 101262174 | Solyc11g045520 |
| ***NRAMP1*** | TGGCCAATTTATCATGCAAGGATT | GCTCCTGACGATCCTCCAAT | 543868 | Solyc11g018530 |
| ***NRAMP3*** | TTTTGCCCTGATCCCCCTTC | GCTACTAGCCATGATATCACCTTCA | 544257 | Solyc02g092800 |
| ***MYB58*** | AGCTGGGTTATTGAGGTGTGG | GGTGTCTTCTTCTTGTGGGG | 104649494 | Solyc10g005550 |
| ***PEPC*** | GACCCGGGTATTGCAGCTC | CCAGCAATCTGGAGAAGGAGG | 101261166 | Solyc10g007290 |
| ***ERF017*** | TTTTTCCGGGGTTCGATGACT | GGTGATGGTTGTGGTGACGA | 101253257 | Solyc12g009240 |
| ***PECTINESTERASE*** | CCTCTACGTCCACTCACTTCG | GAACAACAGCTGCATTACCAAAAA | 101260941 | Solyc06g009190 |
| ***REVEILLE8*** | CCCGGACTTTGAACCCATTAAAAA | ACCACCTGTAGGAAGACCGA | 101253545 | Solyc10g084370 |
| ***FRO6*** | CAGCCTTCATTGGAGGAGGG | ACATCCTTTGAAGCCAGGGG | 101246763 | Solyc01g102610 |
| ***JAR1**** | GCAAATTCTCCAGTCGGCCT | ACGATATAAACCTGCGAAATTGGT | 101262053 | Solyc10g011660 |
| ***Ferritin-1*** | AACGTCCATGCTGTAGCCTC | CCATGTCCTTGGCCAACTCT | 104647958 | Solyc06g050980 |
| ***ChlH*** | GAACCTCAGGAAGGATGGCA | ACAACGTACGTACCTGAGCA | 101244176 | Solyc04g015750 |
| ***CHLN*** | TGCTCTGGAGGAGTGAGTGA | AGACACACAAATAGGACACACTGA | 101248619 | Solyc01g100490 |
| ***OPT3*** | GTGGGGCTTGTTGTTTGCAT | TGTCATATCCGGGTTGCTGATT | 101265194 | Solyc11g012700 |

*This primer pair amplifies every gene transcript variant.

**Table S3.** Experimental validation of a subset of genes regulated by phytoplasma-infection and/or Fe-starvation. Shown are the log2 fold-change values from RNA-seq and qRT-PCR for each gene in the three comparisons: phytoplasma-infected versus healthy Fe-sufficient plants (I/+Fe vs H/+Fe), healthy Fe-starved versus Fe-sufficient (H/-Fe vs H/+Fe) and phytoplasma-infected Fe-sufficient versus Fe-starved plants (I/+Fe vs H/-Fe).

|  |  |  | **LOG_2_(FOLD-CHANGE)** | |
| --- | --- | --- | --- | --- |
| **GENE** | **DESCRIPTION** | **COMPARISON** | **RNA-seq** | **qRT-PCR** |
| Solyc12g009240 | Ethylene-responsive transcription factor 17  (*ERF017*) | I/+Fe vs H/+Fe | 3,49 | 3,51 |
|  |  | H/-Fe vs H/+Fe | 1,23 | 1,15 |
|  |  | I/+Fe vs H/-Fe | 2,27 | 2,36 |
| Solyc06g009190 | Pectinesterase | I/+Fe vs H/+Fe | 2,77 | 2,46 |
|  |  | H/-Fe vs H/+Fe | 0,77 | 0,30 |
|  |  | I/+Fe vs H/-Fe | 2,00 | 2,16 |
| Solyc10g084370 | MYB transcription factor  (*REVEILLE 8*) | I/+Fe vs H/+Fe | -8,27 | -8,22 |
|  |  | H/-Fe vs H/+Fe | -2,10 | -2,02 |
|  |  | I/+Fe vs H/-Fe | -6,17 | -6,19 |
| Solyc01g102610 | Ferric reduction oxidase 6  (*FRO6*) | I/+Fe vs H/+Fe | -5,41 | -5,12 |
|  |  | H/-Fe vs H/+Fe | -2,45 | -2,20 |
|  |  | I/+Fe vs H/-Fe | -2,96 | -2,92 |
| Solyc10g011660 | Jasmonic acid-amido synthetase  (*JAR1*) | I/+Fe vs H/+Fe | 0,03 | -0,29 |
|  |  | H/-Fe vs H/+Fe | 0,81 | 0,56 |
|  |  | I/+Fe vs H/-Fe | -0,78 | -0,85 |
| Solyc02g092800 | *NRAMP3* | I/+Fe vs H/+Fe | 0,27 | -0,16 |
|  |  | H/-Fe vs H/+Fe | 1,00 | 0,73 |
|  |  | I/+Fe vs H/-Fe | -0,73 | -0,90 |
| Solyc06g050980 | Ferritin-1 | I/+Fe vs H/+Fe | -0,20 | -1,69 |
|  |  | H/-Fe vs H/+Fe | -2,81 | -2,62 |
|  |  | I/+Fe vs H/-Fe | 2,61 | 0,93 |
| Solyc04g015750 | Magnesium chelatase H subunit  (*ChlH*) | I/+Fe vs H/+Fe | -4,07 | -5,10 |
|  |  | H/-Fe vs H/+Fe | -1,20 | -0,89 |
|  |  | I/+Fe vs H/-Fe | -2,86 | -4,21 |
| Solyc01g100490 | Nicotianamine synthase-like  (*CHLN*) | I/+Fe vs H/+Fe | -1,39 | -1,12 |
|  |  | H/-Fe vs H/+Fe | -2,33 | -1,90 |
|  |  | I/+Fe vs H/-Fe | 0,94 | 0,78 |
| Solyc11g012700 | Oligopeptide transporter 3  (*OPT3*) | I/+Fe vs H/+Fe | -0,22 | -0,19 |
|  |  | H/-Fe vs H/+Fe | 2,25 | 2,36 |
|  |  | I/+Fe vs H/-Fe | -2,47 | -2,55 |

**Table S4.** Genes associated with Photosynthesis-Antenna Proteins KEGG pathway (00196) in all pairwise comparisons.

In the I versus H (+Fe) comparison, fold-change is the ratio of I/+Fe FPKM on H/+Fe FPKM; similarly, in the comparison -Fe versus +Fe (H), fold-change is the ratio of H/-Fe FPKM on H/+Fe FPKM, and in I/+Fe versus H/-Fe the ratio is calculated as I/+Fe FPKM on H/-Fe FPKM.

Lhca: Light-harvesting chlorophyll protein complexes associated to the Photosystem I. Lchb: Light-harvesting chlorophyll protein complexes associated to the Photosystem II. In bold, DEGs specific for the indicated pairwise comparison. Contra-regulated genes in Infected and Fe-deficient samples are underlined. In bold italic, one Gene ID that NCBI associates to different genes annotated in the Solgenomics ITAG3.0 assembly. Contra-regulated genes in Infected and Fe-deficient samples are underlined. Total FPKM corresponds to the sum of FPKM expression of the corresponding gene in the two compared conditions.

| Ortholog group | Gene Name | NCBI Gene ID | Fold-change | direction | Total FPKM |
| --- | --- | --- | --- | --- | --- |
| **I versus H (+Fe)** | | | | | |
| Lhca2 | **Solyc10g006230** | 101264376 | 3.2 | DOWN | 1097.3 |
| Lhca2 | Solyc12g009200 | 101252151 | 2.2 | DOWN | 35.3 |
| Lhca3 | **Solyc12g011280** | 101265617 | 3.2 | DOWN | 105.5 |
| Lhca4 | **Solyc10g007690** | 101253628 | 2.6 | DOWN | 1228.4 |
| Lhca4 | **Solyc03g115900** | 101268669 | 2.7 | DOWN | 328.0 |
| Lhca4 | Solyc06g069730 | 101256006 | 2.0 | DOWN | 13.7 |
| Lhcb1 | **Solyc02g070940** | ***101264784*** | 8.1 | DOWN | 921.9 |
| Lhcb1 | **Solyc03g005760** | 101267774 | 2.7 | DOWN | 885.6 |
| Lhcb1 | **Solyc02g071010** | ***101264784*** | 2.7 | DOWN | 670.1 |
| Lhcb1 | Solyc02g070970 | ***101264784*** | 2.3 | DOWN | 205.7 |
| Lhcb1 | Solyc03g005770 | 101245729 | 2.4 | DOWN | 134.1 |
| Lhcb1 | **Solyc02g070980** | 104645884 | 5.5 | DOWN | 72.9 |
| Lhcb2 | **Solyc07g047850** | 543975 | 6.0 | DOWN | 781.1 |
| Lhcb2 | **Solyc12g006140** | 543976 | 7.1 | DOWN | 249.7 |
| Lhcb3 | **Solyc12g011450** | 101243766 | 3.9 | DOWN | 740.0 |
| Lhcb3 | **Solyc07g063600** | 101268123 | 2.0 | DOWN | 269.9 |
| Lhcb4 | **Solyc09g014520** | 101249002 | 2.4 | DOWN | 1076.2 |
| Lhcb5 | **Solyc06g063370** | 101266527 | 2.6 | DOWN | 645.4 |
| Lhcb6 | **Solyc01g105030** | 101256629 | 2.3 | DOWN | 364.8 |
| Lhcb6 | **Solyc01g105050** | 101256131 | 2.2 | DOWN | 87.4 |
| **-Fe versus +Fe (H)** | | | | | |
| Lhca1 | **Solyc05g056070** | 544310 | 1.9 | UP | 907.3 |
| Lhca1 | **Solyc05g056050** | 101253380 | 1.9 | UP | 219.3 |
| Lhca2 | Solyc12g009200 | 101252151 | 2.1 | DOWN | 35.7 |
| Lhca4 | Solyc06g069730 | 101256006 | 1.9 | UP | 26.3 |
| Lhcb1 | Solyc02g070970 | ***101264784*** | 2.5 | UP | 499.4 |
| Lhcb1 | Solyc03g005770 | 101245729 | 1.7 | UP | 254.2 |
| Lhcb1 | **Solyc03g005780** | 108491835 | 3.0 | UP | 246.5 |
| Lhcb1 | **Solyc02g070950** | ***101264784*** | 3.2 | UP | 221.4 |
| **I/+Fe versus H/-Fe** | | | | | |
| Lhca1 | Solyc05g056070 | 544310 | 2.4 | DOWN | 844.6 |
| Lhca1 | Solyc05g056050 | 101253380 | 2.7 | DOWN | 195.6 |
| Lhca2 | Solyc10g006230 | 101264376 | 2.6 | DOWN | 950.9 |
| Lhca3 | Solyc12g011280 | 101265617 | 2.5 | DOWN | 88.3 |
| Lhca4 | Solyc10g007690 | 101253628 | 3.1 | DOWN | 1403.4 |
| Lhca4 | Solyc03g115900 | 101268669 | 3.8 | DOWN | 425.8 |
| Lhca4 | Solyc06g069730 | 101256006 | 3.7 | DOWN | 21.7 |
| Lhcb1 | **Solyc02g071030** | ***101264784*** | 3.2 | DOWN | 1823.1 |
| Lhcb1 | Solyc03g005760 | 101267774 | 4.8 | DOWN | 1392.0 |
| Lhcb1 | Solyc02g071010 | ***101264784*** | 4.3 | DOWN | 956.9 |
| Lhcb1 | Solyc02g070940 | ***101264784*** | 7.2 | DOWN | 825.2 |
| Lhcb1 | Solyc02g070970 | ***101264784*** | 5.7 | DOWN | 417.6 |
| Lhcb1 | Solyc03g005780 | 108491835 | 3.4 | DOWN | 239.6 |
| Lhcb1 | Solyc02g070950 | ***101264784*** | 4.2 | DOWN | 208.7 |
| Lhcb1 | Solyc03g005770 | 101245729 | 4.0 | DOWN | 199.3 |
| Lhcb1 | Solyc02g070980 | 104645884 | 7.1 | DOWN | 90.2 |
| Lhcb1 | **Solyc02g070990** | 101266182 | 2.0 | DOWN | 64.5 |
| Lhcb2 | Solyc07g047850 | 543975 | 4.7 | DOWN | 630.7 |
| Lhcb2 | Solyc12g006140 | 543976 | 7.4 | DOWN | 258.7 |
| Lhcb3 | Solyc12g011450 | 101243766 | 4.7 | DOWN | 857.4 |
| Lhcb3 | Solyc07g063600 | 101268123 | 2.9 | DOWN | 351.0 |
| Lhcb4 | Solyc09g014520 | 101249002 | 2.0 | DOWN | 931.2 |
| Lhcb5 | Solyc06g063370 | 101266527 | 2.7 | DOWN | 669.9 |
| Lhcb6 | Solyc01g105030 | 101256629 | 2.4 | DOWN | 379.9 |
| Lhcb6 | Solyc01g105050 | 101256131 | 2.2 | DOWN | 87.8 |

**Table S5.** Genes associated with ‘Porphyrin and chlorophyll metabolism’ KEGG pathway (00860) in all pairwise comparisons.

In the I/+Fe versus H/+Fe comparison, fold-change is the ratio of I/+Fe FPKM on H/+Fe FPKM; similarly, in the comparison H/-Fe versus H/+Fe, fold-change is the ratio of H/-Fe FPKM on H/+Fe FPKM, and in I/+Fe versus H/-Fe the ratio is calculated as I/+Fe FPKM on H/-Fe FPKM.

Total FPKM corresponds to the sum of FPKM expression of the corresponding gene in the two compared conditions.

In bold, DEGs specific for the indicated pairwise comparison. Contra-regulated genes in Infected and Fe-deficient samples are underlined.

| Gene Name | NCBI Gene ID | Gene description  KEGG \| NCBI RefSeq | Fold-  change | direction | Total  FPKM |
| --- | --- | --- | --- | --- | --- |
| **I versus H (+Fe)** | | | | | |
| Solyc10g077040 | 101257518 | magnesium-protoporphyrin IX monomethyl ester (oxidative) cyclase [EC:1.14.13.81] \| at103; putative magnesium-protoporphyrin monomethyl ester cyclase | 4.7 | DOWN | 258.2 |
| Solyc04g015750 | 101244176 | magnesium chelatase subunit H [EC:6.6.1.1] \| magnesium-chelatase subunit ChlH, chloroplastic | 16.7 | DOWN | 221.7 |
| Solyc04g076870 | 101266935 | glutamyl-tRNA reductase [EC:1.2.1.70] \| glutamyl-tRNA reductase 1, chloroplastic | 2.0 | DOWN | 156.2 |
| **Solyc03g115980** | 101262299 | geranylgeranyl diphosphate/geranylgeranyl-bacteriochlorophyllide a reductase [EC:1.3.1.83 / 1.3.1.11] \| geranylgeranyl diphosphate reductase, chloroplastic GGR;CHLP | 2.7 | DOWN | 140.8 |
| Solyc04g063240 | 101252980 | magnesium dechelatase [EC:4.99.1.10] \| protein STAY-GREEN LIKE, chloroplastic | 3.2 | DOWN | 71.3 |
| **Solyc11g012850** | 101244441 | chlorophyllide a oxygenase [EC:1.14.13.122] \| chlorophyllide a oxygenase, chloroplastic | 2.6 | DOWN | 31.8 |
| Solyc06g060310 | 101261422 | chlorophyllide a oxygenase [EC:1.14.13.122] \| chlorophyllide a oxygenase, chloroplastic | 5.6 | DOWN | 20.6 |
| Solyc12g005300 | 101263579 | chlorophyllase [EC:3.1.1.14] \| chlorophyllase-2, chloroplastic | 3.7 | DOWN | 19.8 |
| **Solyc07g024000** | 101258872 | chlorophyll(ide) b reductase [EC:1.1.1.294] \| probable chlorophyll(ide) b reductase NYC1, chloroplastic isoform X2 | 1.7 | UP | 94.4 |
| Solyc12g013710 | 101248079 | protochlorophyllide reductase [EC:1.3.1.33] \| protochlorophyllide reductase-like | 3.6 | UP | 92.8 |
| Solyc01g106390 | 101252440 | glutamyl-tRNA reductase [EC:1.2.1.70] \| glutamyl-tRNA reductase 1, chloroplastic-like | 1.9 | UP | 62.9 |
| Solyc10g006900 | 101244717 | protochlorophyllide reductase [EC:1.3.1.33] \| light dependent NADH:protochlorophyllide | 2.4 | UP | 53.2 |
| Solyc06g053980 | 101258376 | chlorophyllase [EC:3.1.1.14] \| chlorophyllase-2, chloroplastic-like | 2.9 | UP | 41.6 |
| **-Fe versus +Fe (H)** | | | | | |
| Solyc10g077040 | 101257518 | magnesium-protoporphyrin IX monomethyl ester (oxidative) cyclase [EC:1.14.13.81] \| at103, putative magnesium-protoporphyrin monomethyl ester cyclase | 1.9 | DOWN | 328.5 |
| Solyc04g015750 | 101244176 | magnesium chelatase subunit H [EC:6.6.1.1] \| magnesium-chelatase subunit ChlH, chloroplast | 2.3 | DOWN | 299.9 |
| Solyc04g076870 | 101266935 | glutamyl-tRNA reductase [EC:1.2.1.70] \| glutamyl-tRNA reductase 1, chloroplastic | 2.0 | DOWN | 157.4 |
| Solyc04g063240 | 101252980 | magnesium dechelatase [EC:4.99.1.10] \| protein STAY-GREEN LIKE, chloroplastic | 1.9 | DOWN | 82.5 |
| Solyc01g106390 | 101252440 | glutamyl-tRNA reductase [EC:1.2.1.70] \| glutamyl-tRNA reductase 1, chloroplastic-like | 2.3 | DOWN | 30.8 |
| Solyc12g005300 | 101263579 | chlorophyllase [EC:3.1.1.14] \| chlorophyllase-2, chloroplastic | 1.8 | DOWN | 24.0 |
| **Solyc01g086650** | 101261158 | uroporphyrin-III C-methyltransferase [EC:2.1.1.107] \| siroheme synthase | 1.8 | DOWN | 10.2 |
| **Solyc07g054210** | 543647 | protochlorophyllide reductase [EC:1.3.1.33] \| POR2; protochlorophyllide reductase | 1.8 | UP | 148.9 |
| Solyc12g013710 | 101248079 | protochlorophyllide reductase [EC:1.3.1.33] \| protochlorophyllide reductase-like | 4.5 | UP | 110.4 |
| Solyc10g006900 | 101244717 | protochlorophyllide reductase [EC:1.3.1.33] \| light dependent NADH:protochlorophyllide | 3.3 | UP | 66.6 |
| Solyc06g060310 | 101261422 | chlorophyllide a oxygenase [EC:1.14.13.122] \| chlorophyllide a oxygenase, chloroplas | 1.8 | UP | 47.9 |
| Solyc06g053980 | 101258376 | chlorophyllase [EC:3.1.1.14] \| chlorophyllase-2, chloroplastic-like | 2.8 | UP | 40.8 |
| **I/+Fe versus H/-Fe** | | | | | |
| Solyc10g077040 | 101257518 | magnesium-protoporphyrin IX monomethyl ester (oxidative) cyclase [EC:1.14.13.81] \| at103, putative magnesium-protoporphyrin monomethyl ester cyclase | 2.6 | DOWN | 160.6 |
| Solyc07g054210 | 543647 | protochlorophyllide reductase [EC:1.3.1.33] \| POR2; protochlorophyllide reductase | 1.9 | DOWN | 144.8 |
| Solyc03g115980 | 101262299 | geranylgeranyl diphosphate/geranylgeranyl-bacteriochlorophyllide a reductase [EC:1.3.1.83 / 1.3.1.11] \| geranylgeranyl diphosphate reductase, chloroplastic GGR;CHLP | 2.5 | DOWN | 135.7 |
| Solyc04g015750 | 101244176 | magnesium chelatase subunit H [EC:6.6.1.1] \| magnesium-chelatase subunit ChlH, chlor | 7.3 | DOWN | 103.2 |
| Solyc04g063240 | 101252980 | magnesium dechelatase [EC:4.99.1.10] \| protein STAY-GREEN LIKE, chloroplastic | 1.7 | DOWN | 45.2 |
| Solyc06g060310 | 101261422 | chlorophyllide a oxygenase [EC:1.14.13.122] \| chlorophyllide a oxygenase, chloroplast | 9.8 | DOWN | 33.6 |
| Solyc11g012850 | 101244441 | chlorophyllide a oxygenase [EC:1.14.13.122] \| chlorophyllide a oxygenase, chloroplastic | 2.0 | DOWN | 26.4 |
| Solyc12g005300 | 101263579 | chlorophyllase [EC:3.1.1.14] \| chlorophyllase-2, chloroplastic | 2.0 | DOWN | 12.7 |
| Solyc07g024000 | 101258872 | chlorophyll(ide) b reductase [EC:1.1.1.294] \| probable chlorophyll(ide) b reductase NYC1, chloroplastic isoform X2 | 1.8 | UP | 93.7 |
| Solyc01g106390 | 101252440 | glutamyl-tRNA reductase [EC:1.2.1.70] \| glutamyl-tRNA reductase 1, chloroplastic-like | 4.5 | UP | 50.5 |

**Table S6.** Genes associated with Carotenoid Biosynthesis KEGG pathway (00906) in the pairwise comparisons.

In the I/+Fe versus H/+Fe comparison, fold-change is the ratio of I/+Fe FPKM on H/+Fe FPKM; similarly, in the comparison H/-Fe versus H/+Fe, fold-change is the ratio of H/-Fe FPKM on H/+Fe FPKM, and in I/+Fe versus H/-Fe the ratio is calculated as I/+Fe FPKM on H/-Fe FPKM.

Total FPKM corresponds to the sum of FPKM expression of the corresponding gene in the two compared conditions. In bold, DEGs specific for the indicated pairwise comparison.

| Gene Name | NCBI Gene ID | Gene description  KEGG \| NCBI RefSeq | Fold-  change | direction | Total  FPKM |
| --- | --- | --- | --- | --- | --- |
| **I/+Fe versus H/+Fe** | | | | | |
| **Solyc08g075490** | 101250535 | 9-cis-epoxycarotenoid dioxygenase [EC:1.13.11.51] \| probable carotenoid cleavage dioxygenase | 4.7 | DOWN | 191.9 |
| **Solyc02g090890** | 544162 | zeaxanthin epoxidase [EC:1.14.15.21] \| ZEP, ZE; zeaxanthin epoxidase, chloroplastic | 3.0 | DOWN | 101.0 |
| **Solyc04g040190** | 544104 | lycopene beta-cyclase [EC:5.5.1.19] \| LCY1, CrtL-1, LCY-B; lycopene beta-cyclase | 3.6 | DOWN | 68.5 |
| Solyc06g036260 | 544133 | beta-carotene 3-hydroxylase [EC:1.14.15.24] \| CrtR-b1; beta-carotene hydroxylase | 19.2 | DOWN | 64.9 |
| Solyc03g031860 | 543988 | 15-cis-phytoene synthase [EC:2.5.1.32] \| Psy1, GTOM5, psy; phytoene synthase 1, chloroplastic | 5.2 | DOWN | 33.2 |
| Solyc02g081330 | 543964 | 15-cis-phytoene synthase [EC:2.5.1.32] \| PSY2; phytoene synthase 2, chloroplastic | 1.6 | DOWN | 28.1 |
| **Solyc10g079480** | 101267662 | lycopene beta-cyclase [EC:5.5.1.19] \| lycopene beta cyclase, chloroplastic | 1.9 | DOWN | 21.4 |
| Solyc04g078900 | 100136887 | (+)-abscisic acid 8'-hydroxylase [EC:1.14.14.137] \| CYP707A1; ABA 8'-hydroxylase | 2.0 | UP | 110.6 |
| **Solyc11g071620** | 543650 | abscisic-aldehyde oxidase [EC:1.2.3.14] \| AO1, TAO1; aldehyde oxidase | 1.7 | UP | 29.2 |
| **H/-Fe versus H/+Fe** | | | | | |
| Solyc06g036260 | 544133 | beta-carotene 3-hydroxylase [EC:1.14.15.24] \| CrtR-b1; beta-carotene hydroxylase | 2.8 | DOWN | 83.8 |
| **Solyc08g005610** | 101249565 | (+)-abscisic acid 8'-hydroxylase [EC:1.14.14.137] \| CYP707A2; abscisic acid 8'-hydroxylase | 2.3 | DOWN | 46.1 |
| Solyc03g031860 | 543988 | 15-cis-phytoene synthase [EC:2.5.1.32] \| Psy1, GTOM5, psy; phytoene synthase 1, chloroplastic | 3.6 | DOWN | 35.5 |
| Solyc02g081330 | 543964 | 15-cis-phytoene synthase [EC:2.5.1.32] \| PSY2; phytoene synthase 2, chloroplastic | 3.0 | DOWN | 23.2 |
| **Solyc12g056600** | 100750250 | xanthoxin dehydrogenase [EC:1.1.1.288] \| SlscADH1; short-chain dehydrogenase-reductase | 2.6 | DOWN | 4.2 |
| **Solyc08g016720** | 100316877 | 9-cis-epoxycarotenoid dioxygenase [EC:1.13.11.51] \| NCED2; 9-cis-epoxycarotenoid dioxygenase | 2.1 | DOWN | 2.7 |
| Solyc04g078900 | 100136887 | (+)-abscisic acid 8'-hydroxylase [EC:1.14.14.137] \| CYP707A1; ABA 8'-hydroxylase | 1.6 | UP | 96.5 |
| **Solyc11g071600** | 543652 | abscisic-aldehyde oxidase [EC:1.2.3.14] \| TAO3, AO3; aldehyde oxidase | 1.7 | UP | 18.7 |
| **I/+Fe versus H/-Fe** | | | | | |
| Solyc08g075490 | 101250535 | 9-cis-epoxycarotenoid dioxygenase [EC:1.13.11.51] \| probable carotenoid cleavage dioxygenase | 4.9 | DOWN | 198.7 |
| Solyc02g090890 | 544162 | zeaxanthin epoxidase [EC:1.14.15.21] \| ZEP, ZE; zeaxanthin epoxidase, chloroplastic | 3.2 | DOWN | 106.5 |
| Solyc06g036260 | 544133 | beta-carotene 3-hydroxylase [EC:1.14.15.24] \| CrtR-b1; beta-carotene hydroxylase | 6.9 | DOWN | 25.3 |
| **Solyc03g007960** | 544297 | beta-carotene 3-hydroxylase [EC:1.14.15.24] \| CrtR-b2; beta-carotene hydroxylase | 2.1 | DOWN | 17.7 |
| Solyc08g005610 | 101249565 | (+)-abscisic acid 8'-hydroxylase [EC:1.14.14.137] \| CYP707A2; abscisic acid 8'-hydroxylase | 3.4 | UP | 60.8 |
| Solyc11g071620 | 543650 | abscisic-aldehyde oxidase [EC:1.2.3.14] \| AO1, TAO1; aldehyde oxidase | 1.8 | UP | 28.8 |
| **Solyc07g056570** | 544163 | 9-cis-epoxycarotenoid dioxygenase [EC:1.13.11.51] \| LeNCED1; nine-cis-epoxycarotenoid dioxygenase | 1.8 | UP | 24.1 |
| Solyc02g081330 | 543964 | 15-cis-phytoene synthase [EC:2.5.1.32] \| PSY2; phytoene synthase 2, chloroplastic | 1.8 | UP | 16.4 |
| Solyc12g056600 | 100750250 | xanthoxin dehydrogenase [EC:1.1.1.288] \| SlscADH1;short-chain dehydrogenase-reductase | 3.8 | UP | 5.7 |

**Table S7.** Genes associated with Photosynthesis-light reactions KEGG pathway (00195) in all pairwise comparisons.

In the I versus H (+Fe) comparison, fold-change is the ratio of I/+Fe FPKM on H/+Fe FPKM; similarly, in the comparison -Fe versus +Fe (H), fold-change corresponds to the ratio of H/-Fe FPKM on H/+Fe FPKM, and in I/+Fe versus H/-Fe the ratio is calculated as I/+Fe FPKM on H/-Fe FPKM. Total FPKM corresponds to the sum of FPKM expression of the corresponding gene in the two compared conditions. In bold, DEGs specific for the indicated pairwise comparison.

| Gene Name | NCBI Gene ID | Gene description  KEGG \| NCBI RefSeq | Fold-  change | direction | Total  FPKM |
| --- | --- | --- | --- | --- | --- |
| **I versus H (+Fe)** | | | | | |
| **Solyc07g066310** | 778297 | photosystem II 10kDa protein \| PSBR; PSII polypeptide | 2.0 | DOWN | 8353.3 |
| **Solyc11g051170** | 101265249 | photosystem I subunit XI \| photosystem I reaction center subunit XI, chloroplastic | 1.9 | DOWN | 5134.8 |
| **Solyc06g072540** | 101268297 | photosystem I subunit PsaN \| photosystem I reaction center subunit | 2.3 | DOWN | 4944.3 |
| **Solyc04g082010** | 544053 | plastocyanin \| PETE; plastocyanin, chloroplastic | 1.9 | DOWN | 1296.6 |
| **Solyc06g054260** | 543978 | photosystem I subunit II \| PSI-D, psaD; photosystem I reaction center subunit II, chloroplastic | 1.9 | DOWN | 1279.6 |
| **Solyc08g006930** | 101255222 | photosystem I subunit X \| photosystem I reaction center subunit psaK, chloroplastic | 2.5 | DOWN | 1071.9 |
| **Solyc06g074200** | 101254806 | photosystem I subunit PsaO \| photosystem I subunit O | 2.0 | DOWN | 1070.0 |
| **Solyc10g077120** | 101259494 | photosystem II PsbY protein \| photosystem II core complex proteins psbY, chloroplast | 1.6 | DOWN | 854.7 |
| **Solyc08g013670** | 101268297 | photosystem I subunit PsaN \| photosystem I reaction center subunit | 3.4 | DOWN | 760.7 |
| **Solyc02g083810** | 101261284 | ferredoxin--NADP+ reductase [EC:1.18.1.2] \| ferredoxin--NADP reductase, leaf-type isozyme, chloroplastic | 1.9 | DOWN | 446.4 |
| **Solyc12g005630** | 101243864 | cytochrome b6-f complex iron-sulfur subunit [EC:1.10.9.1] \| cytochrome b6-f complex | 1.8 | DOWN | 434.0 |
| **Solyc06g082950** | 101265555 | photosystem I subunit XI \| photosystem I reaction center subunit XI, chloroplastic | 1.6 | DOWN | 427.7 |
| **Solyc02g080540** | 101253342 | F-type H+-transporting ATPase subunit gamma \| ATP synthase gamma chain, chloroplasti | 1.7 | DOWN | 316.3 |
| Solyc10g075160 | 101265784 | ferredoxin \| ferredoxin | 4.1 | DOWN | 268.6 |
| **Solyc06g066000** | 109120519 | F-type H+-transporting ATPase subunit b \| ATP synthase subunit b', chloroplastic-like | 1.7 | DOWN | 147.7 |
| Solyc06g060340 | 101260830 | photosystem II 22kDa protein \| psbS, CP22; photosystem II subunit S | 4.1 | DOWN | 111.9 |
| **Solyc12g044280** | 101244751 | photosystem I subunit VI \| photosystem I reaction center subunit VI, chloroplastic-l | 1.7 | DOWN | 71.5 |
| **Solyc06g065990** | 101263124 | F-type H+-transporting ATPase subunit b \| ATP synthase subunit b', chloroplastic | 1.8 | DOWN | 65.9 |
| Solyc09g064500 | 101245880 | photosystem II 13kDa protein \| photosystem II reaction center Psb28 protein | 1.7 | DOWN | 49.1 |
| Solyc11g006910 | 101266472 | ferredoxin \| ferredoxin, root R-B2-like | 2.4 | DOWN | 9.5 |
| **-Fe versus +Fe (H)** | | | | | |
| Solyc10g075160 | 101265784 | ferredoxin \| ferredoxin | 3.0 | DOWN | 286.7 |
| Solyc06g060340 | 101260830 | photosystem II 22kDa protein \| (RefSeq) psbS, CP22; photosystem II subunit S | 3.6 | DOWN | 115.4 |
| Solyc09g064500 | 101245880 | photosystem II 13kDa protein \| photosystem II reaction center Psb28 protein | 2.3 | DOWN | 44.4 |
| **Solyc03g114930** | 101259227 | photosystem II oxygen-evolving enhancer protein 2 \| psbP-like protein 1, chloroplast | 1.9 | DOWN | 12.3 |
| Solyc11g006910 | 101266472 | ferredoxin \| ferredoxin, root R-B2-like | 2.3 | DOWN | 9.6 |
| **I/+Fe versus H/-Fe** | | | | | |
| Solyc07g066310 | 778297 | photosystem II 10kDa protein \| (RefSeq) PSBR; PSII polypeptide | 2.2 | DOWN | 8955.4 |
| Solyc11g051170 | 101265555 | photosystem I subunit XI \| (RefSeq) photosystem I reaction center subunit XI, chloroplastic | 1.6 | DOWN | 4610.9 |
| Solyc06g054260 | 543978 | photosystem I subunit II \| (RefSeq) PSI-D, psaD; photosystem I reaction center subunit II, ch | 1.8 | DOWN | 1240.7 |
| Solyc06g074200 | 101254806 | photosystem I subunit PsaO \| (RefSeq) photosystem I subunit O | 2.3 | DOWN | 1152.6 |
| **Solyc07g044860** | 544077 | photosystem II oxygen-evolving enhancer protein 2 \| (RefSeq) PSBP, OEE2, psbX; photosystem II | 1.7 | DOWN | 1000.9 |
| Solyc08g006930 | 101255222 | photosystem I subunit X \| (RefSeq) photosystem I reaction center subunit psaK, chloroplastic | 1.9 | DOWN | 878.3 |
| **Solyc06g082940** | 101265249 | photosystem I subunit XI \| (RefSeq) photosystem I reaction center subunit XI, chloroplastic | 1.6 | DOWN | 835.2 |
| Solyc08g013670 | 101268297 | photosystem I subunit PsaN \| (RefSeq) photosystem I reaction center subunit | 2.1 | DOWN | 539.5 |
| Solyc06g082950 | 101265555 | photosystem I subunit XI \| (RefSeq) photosystem I reaction center subunit XI, chloroplastic | 1.9 | DOWN | 476.8 |
